# Comparative Evaluation of Myography Techniques for Prosthetic Hand Control

**DOI:** 10.7759/cureus.114966

**Published:** 2026-08-22

**Authors:** Dennis K Yusuf, Nneamaka H Okeke, Purity T Gandu, Michael U Uzorchukwu, Yash K Patel, Chiahanam J Nwobodo, Enyichukwu T Ogbonna, Godwin Umbugadu, Emmanuel I Otumala, Idris Amodu, Hope N Gandu

**Affiliations:** 1 Electronics and Computer Engineering, Lagos State University, Lagos, NGA; 2 General Medicine, Glangwili General Hospital, Wales, GBR; 3 General Surgery, Tambov State University, Tambov, RUS; 4 Internal Medicine, University of Abuja, Abuja, NGA; 5 Medicine, Baroda Medical College, Vadodara, IND; 6 Medicine, Nnamdi Azikiwe University Teaching Hospital, Nnewi, NGA; 7 Electrical and Electronics Engineering, Michael Okpara University of Agriculture, Umudike, NGA; 8 Electrical and Electronics Engineering, Abubakar Tafawa Balewa University, Bauchi, NGA; 9 Electrical and Electronics Engineering, University of Ilorin, Ilorin, NGA; 10 Life Sciences, University of Westminster, London, GBR

**Keywords:** biofeedback, emg, fmg, hand prosthetics, myography, omg, smg

## Abstract

This review evaluates electromyography (EMG), force myography (FMG), sonomyography (SMG), and optical myography (OMG) for prosthetic hand control, comparing the modalities across signal propagation, signal accuracy, noise resistance, real-time processing, suitability for prosthetic control, and relative cost and system complexity. Upper limb loss affects a substantial and growing population worldwide, yet prosthesis abandonment remains common, in part because of limitations in control, functionality, and intuitive operation. Surface EMG is the most clinically established modality and supports real-time control, but its performance can be affected by noise, electrode-related factors, and reduced classification stability across recording sessions. FMG, including optical fiber implementations, has demonstrated high gesture classification performance in controlled studies and offers resistance to electromagnetic interference together with relatively simple signal processing; however, reported results vary according to sensor configuration, task, participant population, and validation method. SMG provides access to deformation of both superficial and deeper muscle structures but is limited by transducer position sensitivity and comparatively greater acquisition and processing requirements. OMG provides a noninvasive optical approach, with near-infrared implementations showing potential for wearable sensing, although evidence for long-term prosthetic hand control remains limited. Because the available studies differ substantially in participant populations, gesture sets, sensing configurations, classification algorithms, and validation procedures, their reported performance values cannot be interpreted as direct head-to-head comparisons. Among the approaches reviewed, optical fiber FMG is a promising option for portable prosthetic hand control under the conditions evaluated in existing studies, although current evidence does not establish a universally superior modality.

## Introduction and background

Limb loss, whether resulting from trauma, vascular disease, or other critical conditions, profoundly disrupts a person's ability to carry out the ordinary activities of daily living. Traumatic amputation alone affects tens of millions of people worldwide, and its global burden has increased over recent decades [1]. For those affected, a prosthetic device is often the primary route back to independence, yet a substantial proportion of users remain dissatisfied with their devices, commonly citing limited control, poor functionality, and a lack of intuitive operation [2].

Prosthetic technology has advanced enormously, from early wooden replacements to lightweight modern devices and, more recently, to robotic limbs equipped with biosignal-based control systems capable of reproducing increasingly complex movements of the missing limb. Such systems acquire physiological signals associated with the user's movement intention and translate them into commands that drive an appropriate prosthetic action. Myography provides one established means of acquiring these control signals.

A myograph is any instrument used to measure the activity, classically the force, produced by a contracting muscle, while myography refers broadly to the recording and analysis of muscle activity or contraction-related signals. A range of myography techniques has been developed to detect, measure, process, and analyze the signals generated during muscular activity. As the underlying technology has matured, the measurement and interpretation of these signals have served not only to characterize muscle behavior and identify abnormalities but also to build progressively more capable human-machine interfaces [3].

Among the earliest examples of myography applied to prosthetic control was the French Electric Hand, an upper limb prosthetic device developed in the 1950s that was driven by a pneumatic force transducer responding to muscle activity [4]. This early use of mechanical muscle force sensing for limb control foreshadowed the modern field, and in the decades that followed, myography was refined into the more robust electrical, mechanical, and optical techniques available today, particularly for health and rehabilitation applications. Its relevance is difficult to overstate: myography now underpins much of the effort to restore lost limb function through prosthetics. Four modalities are considered in this review, each capturing muscle activity in a different way: electromyography (EMG) detects the electrical signals generated during muscle activation; force myography (FMG) senses the mechanical pressure or stiffness changes produced as muscles contract; sonomyography (SMG) uses ultrasound echoes to detect internal muscle deformation; and optical myography (OMG) uses light-based measurements or imaging to detect contraction-related changes in the muscle or overlying tissue. These signals can then be processed to infer movement intention and provide control inputs to a prosthesis or other human-machine interface.

When attention is narrowed specifically to prosthetic hand devices intended to restore hand and finger function after loss of the hand or wrist, it becomes clear that not every technique is equally suited to the task. Properties such as signal acquisition, signal processing, response speed, portability, system complexity, and cost all bear on whether a given method can convincingly and comfortably replace the lost limb. Each technique carries distinct advantages and limitations, making particular modalities better suited to certain applications and user requirements than others.

Despite considerable progress, selecting an appropriate myography technique for prosthetic control remains challenging because factors such as signal propagation, accuracy, noise resistance, real-time processing, suitability for prosthetic use, and system complexity may favor different approaches under different conditions. This paper reviews four myography modalities selected for detailed evaluation, namely, EMG, FMG, SMG, and OMG, and compares their strengths, limitations, and application-specific suitability for prosthetic hand control. Specifically, this review aims to examine the selected myography modalities, establish their respective advantages and disadvantages, compare their performance across the predefined evaluation criteria, and identify application-dependent considerations that may guide the selection and future development of prosthetic hand control interfaces.

**Figure 1:**
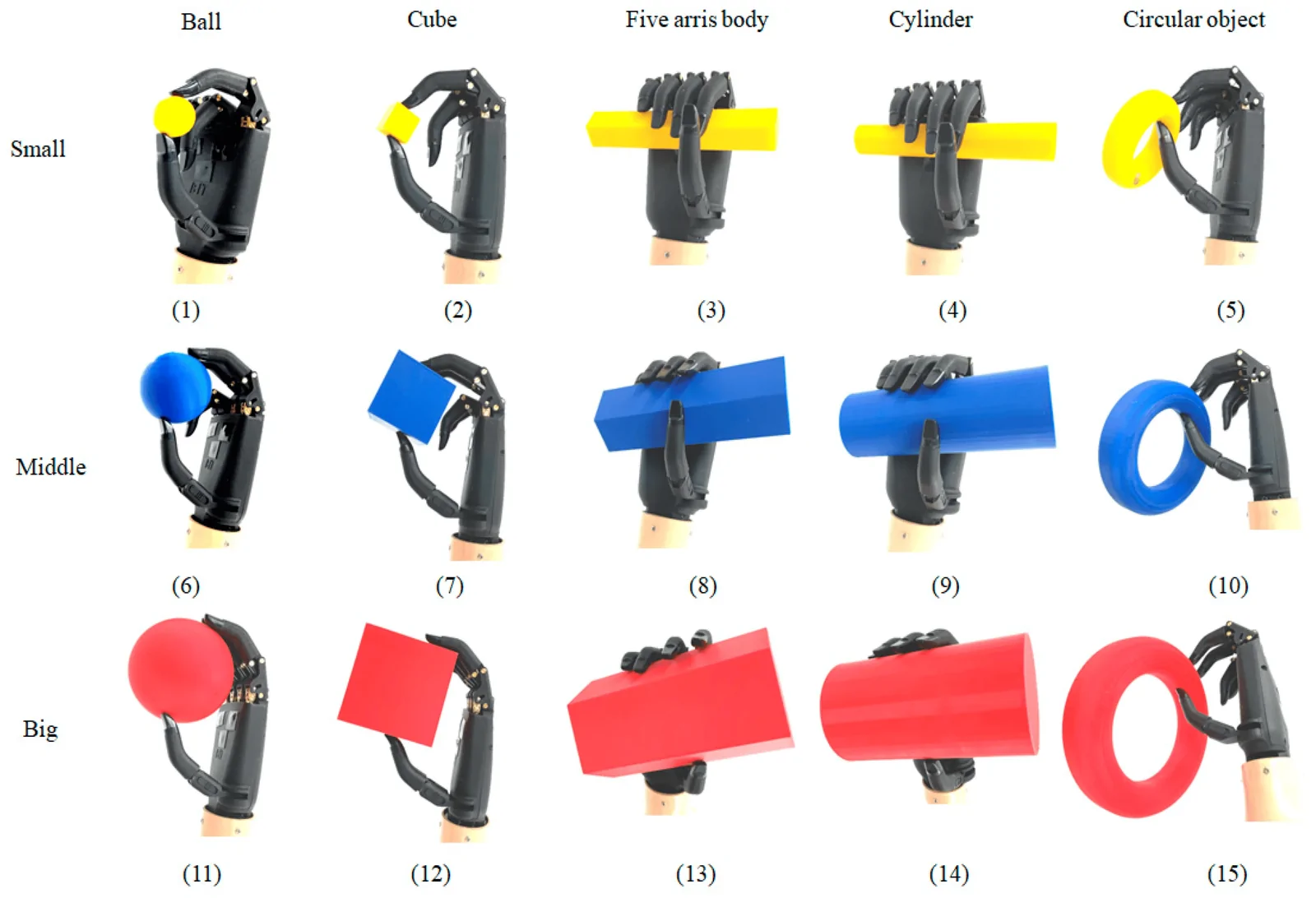
A myoelectric prosthetic hand illustrating adaptive grasp control enabled by myography-based signal acquisition The myoelectric prosthetic hand demonstrates adaptive grasping of objects of different shapes and sizes. Surface electromyography signals are acquired from the user's forearm and processed using pattern recognition to identify grasping intention and control the prosthetic hand. Image Credit: Wang et al. [5]; reproduced under the Creative Commons Attribution (CC BY) license (CC BY 4.0 Deed)

Figure 1 depicts a myoelectric prosthetic hand demonstrating adaptive grasp control enabled by myography-based signal acquisition.

Evaluation criteria

To provide a structured comparison of the four myography modalities selected for detailed evaluation, the following criteria are applied. Signal propagation describes how well each method captures and transmits muscle activity signals. Signal accuracy refers to the precision and reliability of the signals obtained. Noise resistance is the ability to reject external interference and maintain signal integrity. Real-time processing capability concerns the speed and computational efficiency required to drive prosthetic control. Suitability for prosthetic control reflects how readily each method integrates with prosthetic devices and how effectively it controls movement. Relative cost and system complexity consider the sensing hardware, signal conditioning and processing requirements, maintenance burden, and, where applicable, procedural requirements associated with implantation. Because directly comparable published monetary costs are limited across the modalities, this criterion is interpreted qualitatively rather than using absolute price estimates.

This article was previously presented as a poster during the Association of Nigerian Physicians in the Americas (ANPA) Virtual Poster Session on June 7, 2026.

## Review

Literature search and narrative synthesis

This review was conducted as a narrative review of myography-based approaches relevant to prosthetic hand control. For the revised manuscript, updated structured literature searches were conducted in PubMed and IEEE Xplore. The final searches and record exports were completed on August 17, 2026. PubMed searches covered publications from January 1, 2000, through August 16, 2026, whereas IEEE Xplore searches were limited to publication years 2000-2026. Separate searches were conducted for EMG, FMG, SMG, and OMG using combinations of modality-specific terms with terms related to prostheses, upper limb control, hand gesture recognition, and human-machine interfaces. Additional targeted searches were performed for mechanomyography and emerging magnet-based muscle tracking approaches because of their relevance to contemporary prosthetic control research. The complete database-specific search strategies and search settings are provided in Appendix A to improve transparency and reproducibility. Eligibility criteria were organized using a modified Population, Intervention/Concept, Comparator, Outcomes, and Study design (PICOS) framework appropriate to the technology-focused scope of this narrative review; the complete framework is presented in Appendix B. The structured search was limited to PubMed and IEEE Xplore; therefore, relevant studies indexed exclusively in other multidisciplinary databases may not have been retrieved, and this represents a limitation of the review. 

English-language peer-reviewed journal articles and full conference papers were considered when they contributed evidence relevant to sensing principles, signal characteristics, movement classification, real-time implementation, robustness, wearable or prosthetic integration, or practical limitations. Primary experimental studies were prioritized for direct modality-specific evidence, while review articles were used for broader technical context and citation chaining. The framework specifies the eligible participant populations, sensing concepts, outcomes, study designs, publication characteristics, and exclusions. Earlier seminal publications predating 2000 were retained when they provided important historical or foundational information.

Titles and abstracts identified through the updated searches were reviewed for relevance to hand or upper limb prosthetic control. Records retained after title/abstract screening formed the evidence pool for targeted full-text evaluation and citation chaining. Full texts of studies selected to support the principal modality-specific comparisons and performance claims were examined in detail. Studies reporting modality-specific performance were interpreted in the context of their participant populations, experimental tasks, sensing configurations, classifiers, and validation procedures rather than treated as directly comparable quantitative outcomes. The search and screening flow is summarized in Appendix C.

Because the identified studies differed substantially in participant populations, amputation status, sensor configurations, numbers and types of gestures, classification algorithms, validation procedures, and reported outcome measures, quantitative pooling was not considered appropriate. The evidence was therefore synthesized narratively across six predefined domains: signal propagation, signal accuracy, noise resistance, real-time processing, suitability for prosthetic control, and relative cost and system complexity. No meta-analysis, meta-regression, or formal statistical aggregation was performed, and no review-level pooled effect estimates, confidence intervals, or p-values were calculated. To provide structured descriptive aggregation without implying quantitative comparability, participant numbers, task or gesture sets, validation designs, principal performance findings, and measures of dispersion where reported are summarized in Appendix D. Methodological quality of the primary human participant studies contributing direct modality-specific performance or prosthetic control feasibility evidence was assessed using the Mixed Methods Appraisal Tool (MMAT), version 2018 [6]. Studies were classified according to the applicable quantitative non-randomized or quantitative descriptive MMAT category, and the corresponding methodological criteria were rated as "Yes", "No", or "Can't tell" based on information reported in the primary publication. Companion publications arising from the same or closely related experimental cohort were considered together when they contributed overlapping evidence. Consistent with MMAT guidance, criterion-level judgments were not combined into an overall numerical quality score. Instead, the appraisal was used to identify methodological strengths and limitations relevant to interpretation. Applicability to prosthetic hand users was considered separately from the MMAT criteria, with particular attention to participant population, sample size, within-session versus cross-session validation, sensor configuration, experimental task, and completeness of longitudinal evaluation. Engineering-only prototype studies and review articles were retained for relevant technical context but were not subjected to participant-based MMAT criteria when those criteria were not applicable. Study-level appraisal findings are presented in Appendix E and were considered when interpreting the comparative evidence.

Clinical burden of upper limb loss

Upper limb amputation, the loss of the hand and arm responsible for strength, sensation, and fine motor function, represents a profound clinical and functional disability that affects a person's physical capabilities, psychological wellbeing, and social and economic participation. Its scale is substantial and growing. In the United States alone, an estimated 1.6 million people were living with limb loss in 2005, a figure projected to more than double to 3.6 million by 2050 as the population ages and vascular disease becomes more prevalent [7], and the global burden of traumatic amputation has followed a similar upward trend [1]. Upper limb loss arises from diverse causes, including traumatic injury, malignancy, vascular and infectious disease, and congenital difference, with trauma the leading cause, particularly among younger, economically active men in low- and middle-income countries. Because it disproportionately affects people in their working years, its societal and economic impact is amplified.

Compared with lower limb amputation, which primarily compromises mobility, upper limb amputation disrupts the complex motor functions that underpin independence, reducing a person's ability to perform the activities of daily living and frequently necessitating long-term assistance and adaptive strategies. The loss also interrupts the closed loop between the brain and the limb, severing both the efferent pathways that carry motor commands and the afferent pathways that return sensory feedback [8]. Beyond the physical impairment, the psychological burden is considerable, with many individuals experiencing depression, anxiety, phantom limb pain, and disturbances of body image, all of which can reduce quality of life and hinder rehabilitation. For these reasons, a prosthesis is rarely a complete solution, and rejection remains common: surveys of upper limb prosthesis users report mean device rejection rates of roughly a quarter, with dissatisfaction frequently attributed to limited function and unintuitive control [2].

Anatomy and function of the human hand

The difficulty of replacing the hand follows directly from how remarkable it is. The human hand performs three fundamental functions, namely, grasping, manipulation, and fine motor coordination, and it does so through an intricate arrangement of bones, muscles, tendons, ligaments, nerves, and soft tissue. Its dexterity rests on an exceptional number of degrees of freedom: the hand provides 21 and the wrist a further 6, and the thumb, with its unique capacity for opposition, contributes disproportionately to manual dexterity [8]. This combination allows the hand to move fluidly between powerful gripping and delicate precision tasks. The hand is also central to how people sense and act on the world and to social interaction itself, which is part of why its loss is felt so acutely. Any device that aspires to restore hand function must therefore reproduce a wide range of coordinated movements, which sets a demanding benchmark for the control interface.

The need for intuitive prosthetic control

Despite decades of progress, today's upper limb prostheses remain limited, and one of the central engineering challenges is to embed actuators, sensors, and electronics within a device of the same size and weight as the missing limb while still providing control that feels natural. Conventional myoelectric prostheses, which rely on surface EMG, are constrained by signal noise, electrode displacement, and variability in muscle activation, and they often require users to learn non-intuitive control strategies that impose a heavy cognitive load and reduce efficiency during everyday tasks [3,8]. This gap motivates the search for more intuitive control interfaces that integrate smoothly with the user's neuromuscular and cognitive processes. Approaches such as pattern recognition, targeted muscle reinnervation, peripheral nerve interfaces, and brain-computer interfaces all aim to improve signal fidelity and enable more natural control [9]. Within this landscape, myography offers a practical and accessible route to capturing a user's movement intention, and the remainder of this paper examines the four modalities selected for detailed comparison and their suitability for restoring hand function.

EMG

Overview

EMG is a well-established electrodiagnostic technique used to assess the electrical activity of skeletal muscle and contributes to the evaluation of neuromuscular disorders. Beyond its diagnostic applications, EMG is also the most widely adopted myographic modality for upper limb prosthetic control and remains the foundational sensing technology in many commercially available myoelectric prostheses [10]. In prosthetic applications, EMG records the electrical activity generated by muscle fibers during activation. These myoelectric signals can be acquired invasively using needle, fine wire, or implanted electrodes positioned within the muscle or noninvasively using surface electrodes placed on the skin overlying the target muscles. Because invasive approaches involve additional procedural and practical considerations, surface EMG remains the most common approach in conventional commercial prosthetic control [10].

Principle of Operation

**Figure 2:**
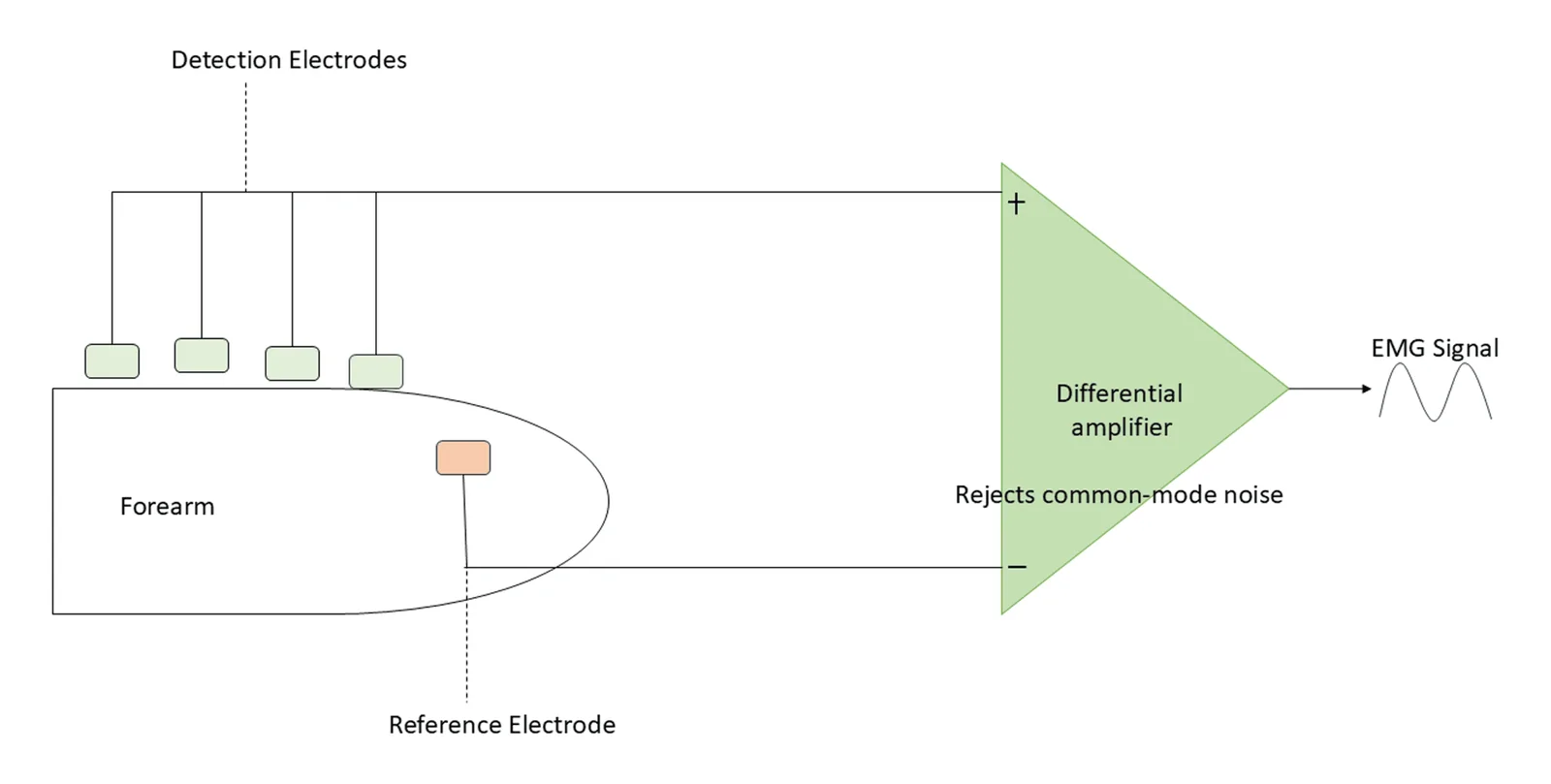
Surface EMG acquisition Multiple detection electrodes placed over the forearm muscle, together with a reference electrode, feed a differential amplifier that rejects common-mode noise and outputs the EMG signal. EMG: electromyography Image Credit: Authors; created using Microsoft PowerPoint (Microsoft Corporation, Redmond, Washington, United States)

When a muscle contracts, the depolarization of its constituent muscle fibers generates small electrical potentials known as motor unit action potentials. EMG captures the summation of these potentials. Surface electrodes detect the field that reaches the skin, while intramuscular electrodes record more locally from within the muscle itself [11]. The recorded amplitude, which typically ranges from a few microvolts up to roughly 10 millivolts, reflects the recruitment and firing rate of the underlying motor units [11]. These very small potentials are amplified and, after processing, translated by a microprocessor into limb movement through the prosthesis actuators, for example, terminal device opening and closing, wrist rotation, or elbow flexion (Figure 2). It is worth noting that conventional myoelectric control is unidirectional: it reads motor intent but does not, by itself, restore sensation to the user, which is why sensory feedback remains an active and separate area of prosthetics research [12].

Signal Propagation and Acquisition

An important practical limitation of surface EMG is its reduced stability across recording sessions. EMG classification performance is known to degrade over time when a classifier is not retrained. In a seven-day study of 10 able-bodied participants and six individuals with transradial amputation, the longitudinal performance of several classification techniques was compared, with artificial neural networks achieving the highest accuracy, followed by linear discriminant analysis [13]. A companion analysis by the same group found that within-day performance remained essentially stable, whereas between-day classification error rose significantly as the interval between the training and testing day increased [14]. The practical implication is that single-session calibration does not hold up well across days, which is one of the main reasons EMG systems require frequent recalibration; collecting training data across multiple days improves robustness but may be impractical for everyday use.

Surface EMG is commonly acquired using a bipolar electrode configuration, in which two closely spaced detection electrodes measure the potential difference over the target muscle, often together with a reference electrode. Differential amplification attenuates interference common to the detection inputs while preserving the differential myoelectric signal [15,16].

Surface EMG is typically acquired over a bandwidth extending to approximately 400-500 Hz, with much of the useful signal energy occurring at lower frequencies [15,16]. Sampling frequencies of at least 1 kHz are therefore commonly used, with rates of approximately 2 kHz providing additional margin for signal acquisition and anti-alias filtering [16]. Because surface EMG signals are relatively low in amplitude, amplification and signal conditioning are required before digital processing. Recorded amplitude and spectral characteristics are influenced by physiological and technical factors, including contraction intensity, muscle fiber depth, subcutaneous tissue thickness, and electrode placement and orientation [11,15,16].

Signal Processing and Noise Resistance

Bioelectric signals are intrinsically difficult to work with because they are susceptible to interference and artefacts from many sources. Surface EMG is influenced by physiological factors such as muscle fatigue, sweating, and changes in skin impedance, as well as by variability between users, all of which make the signal non-stationary and degrade classification accuracy during prolonged use [10,15]. Motion artefacts, which typically appear as high-amplitude, low-frequency disturbances, can be attenuated using appropriate high-pass filtering, although filtering may not eliminate all artefacts. EMG is also contaminated by electrocardiographic (ECG) activity, which is most pronounced when recording from muscles of the upper trunk; because the ECG overlaps the EMG in both amplitude and frequency, it cannot be removed by ordinary filtering, and a common mitigation is to place the recording electrodes as far from the heart as practical [16].

**Figure 3:**
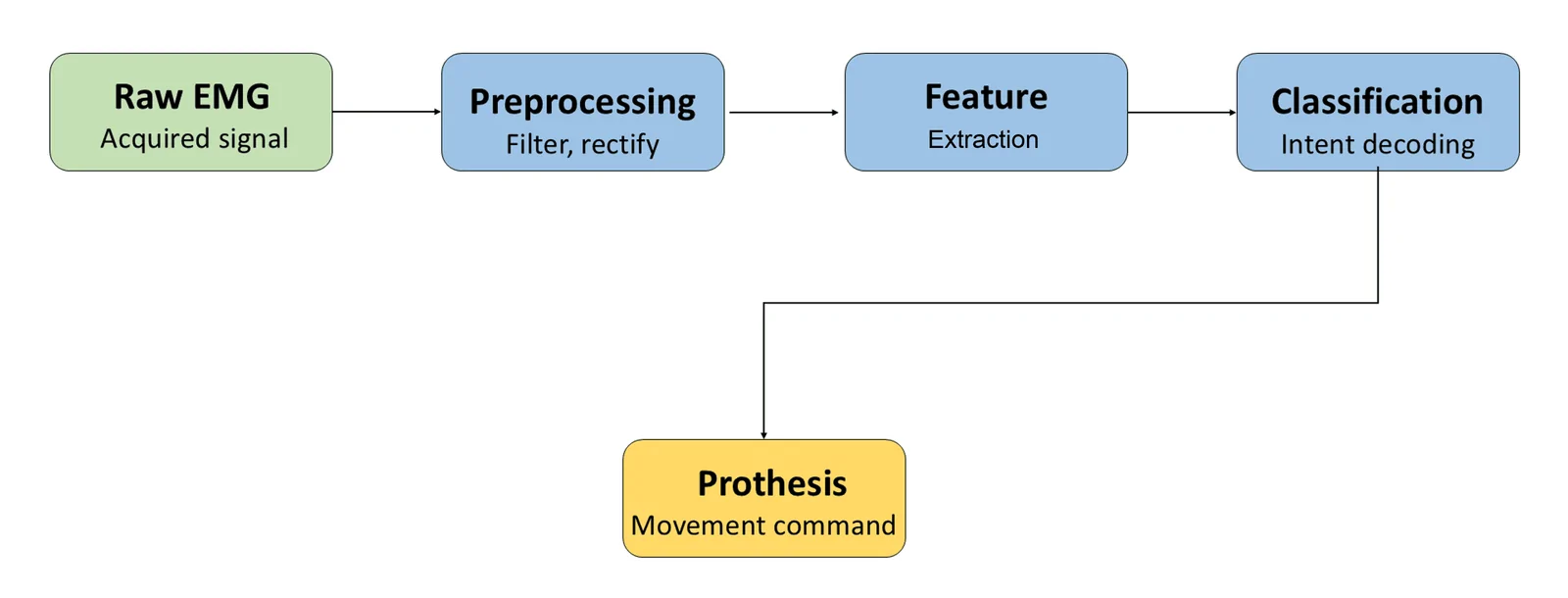
EMG signal processing pipeline The raw EMG signal passes through preprocessing (filtering and rectification), feature extraction, and classification (intent decoding) to produce a prosthesis movement command. EMG: electromyography Image Credit: Authors; created using Microsoft PowerPoint (Microsoft Corporation, Redmond, Washington, United States)

Converting raw EMG into actionable commands therefore involves a multistage pipeline of preprocessing, feature extraction, and classification [10,16] (Figure 3). While this pipeline enables reasonable control, it also adds computational burden and complexity to the system, and it is a recurring theme in the literature that this preprocessing requirement is one of EMG's principal drawbacks relative to mechanically and optically based techniques [10].

Real-Time Processing Capabilities

EMG is valued for its high temporal resolution, which allows muscle activation to be detected rapidly and makes it well suited to real-time control [17]. Studies report that EMG-based systems can achieve control with low latency, generally well within the range considered acceptable for responsive prosthetic use [17]. The tradeoff is that the signal processing required to suppress noise introduces computational overhead, which can extend response time. Pattern recognition and machine learning approaches can support more flexible real-time control, although their performance remains dependent on training conditions and signal stability [10].

Suitability for Prosthetic Control

Invasive approaches can provide greater signal selectivity, but they require electrode insertion or implantation and therefore introduce additional procedural or surgical risks, including infection and foreign body tissue responses. The noninvasive method avoids these problems, but at the cost of reduced signal quality and a greater burden on the user, who must often contract muscles deliberately and consistently to generate detectable, repeatable signals. A further long-standing limitation is the difficulty of achieving intuitive, simultaneous control over multiple degrees of freedom; traditional EMG systems frequently rely on sequential or mode switching strategies that feel unnatural to users, and even pattern recognition approaches struggle to deliver stable, proportional multiple degree of freedom control [10]. Despite these constraints, EMG remains the most clinically established modality and continues to be deployed in commercial multi-articulating devices such as the Ossur i-Limb, which has been evaluated in transradial prosthesis users performing functional daily living tasks [18].

Relative Cost and System Complexity

Surface EMG requires recording electrodes, amplification, signal conditioning, data acquisition hardware, and a processing pipeline for feature extraction and classification [10,16]. These components are technically mature and are already incorporated into commercially available myoelectric prosthetic systems [10,18], although calibration and signal processing requirements add to overall system complexity. Intramuscular or implanted EMG involves greater implementation complexity because electrodes must be positioned within the muscle, making the interface invasive. Thus, surface EMG has moderate relative system complexity, whereas intramuscular approaches involve additional procedural requirements.

FMG

Where EMG reads the electrical signature of muscle contraction, the next technique instead senses its mechanical consequence. FMG exchanges electrodes for force sensors, and with that change comes a markedly simpler signal chain.

Overview

FMG is a noninvasive technique that tracks functional movement by sensing the volumetric changes that occur in a limb as its muscles contract [19]. Rather than measuring the electrical activity of muscle, FMG captures the mechanical consequence of contraction: as a muscle swells and stiffens, it presses outward against the skin, and sensors worn around the limb register that change in pressure or force. This mechanical signal is then decoded into a measurable output that can drive a prosthesis.

The approach is not new. Its lineage runs from the pneumatically driven French Electric Hand of the 1950s [4] through to the soft socket with pneumatic sensors developed by Abboudi and colleagues in the 1990s for multi-finger prosthetic control [20]. Modern FMG has developed into a practical noninvasive approach for human-machine interfaces, with comparatively straightforward signal acquisition and processing [21].

A range of sensing methods can be used to realize FMG, differing mainly in the transducer chosen to detect the volumetric change. Most systems are built on resistive polymer thick film technology using force-sensitive resistors (FSRs), arranged as either a matrix or an array. Other variants use capacitive, textile, piezoelectric, pneumatic, or optical fiber sensors (OFSs), with optical fiber standing out for its flexibility, light weight, and immunity to electromagnetic interference. This section concentrates on the two most widely used: the FSR and the OFS.

Principle of Operation

An FSR is a variable resistor whose resistance falls as the applied force or pressure rises. It is typically built from a conductive polymer pressed between two electrode layers, and it responds electrically to the volumetric changes of the muscle beneath it when placed against the skin. With no pressure applied, the sensor behaves as an open circuit and registers no signal; as the muscle contracts and presses against it, its resistance drops, and a measurable change results.

**Figure 4:**
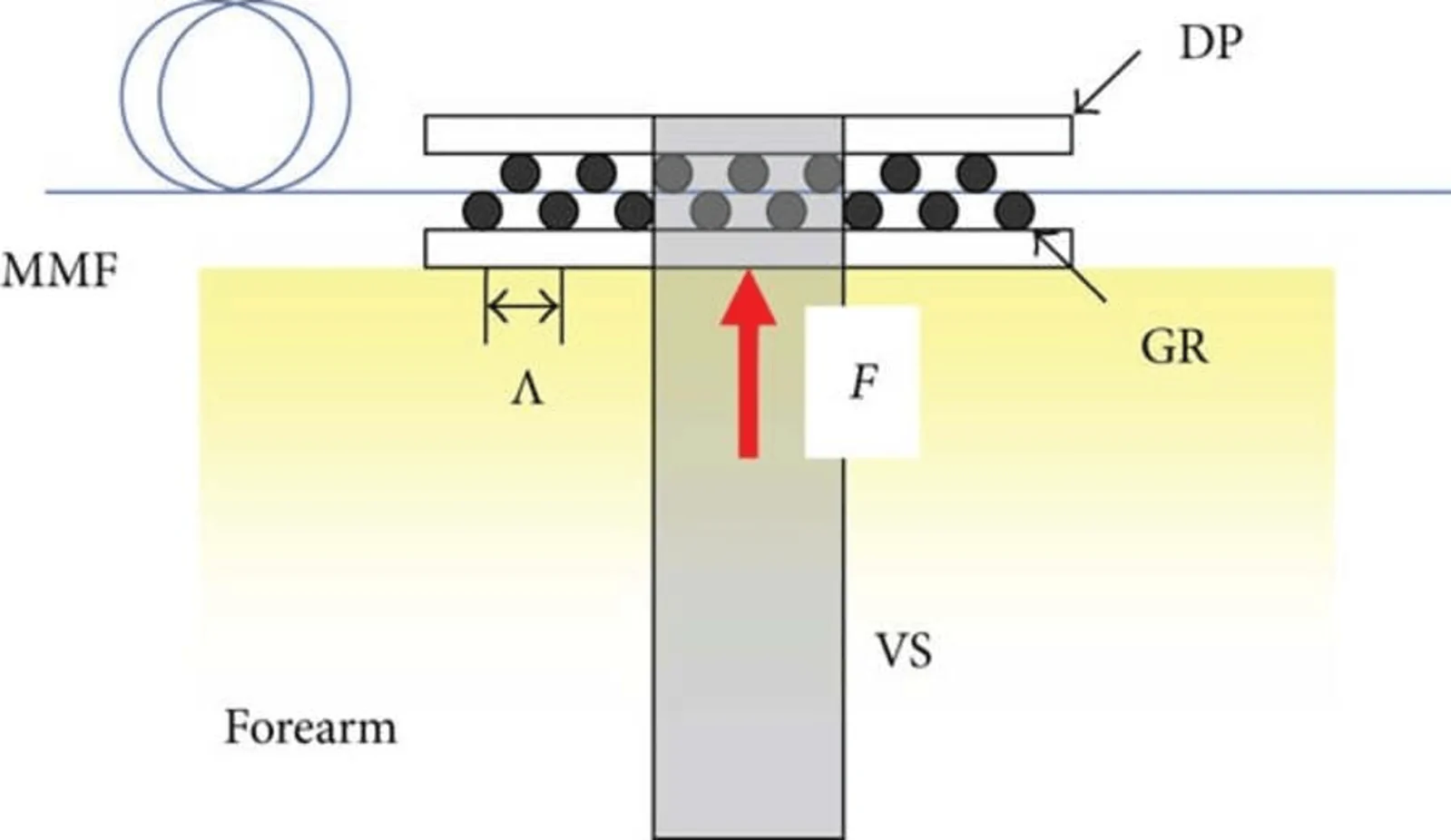
Schematic of the optical fiber force myography transducer MMF: multimode fiber; DP: deformer plate; GR: graphite rods; VS: Velcro strap; Λ: microbending periodicity; F: applied force Image Credit: Fujiwara and Suzuki [22]; reproduced under the Creative Commons Attribution (CC BY) license (CC BY 4.0 Deed)

**Figure 5:**
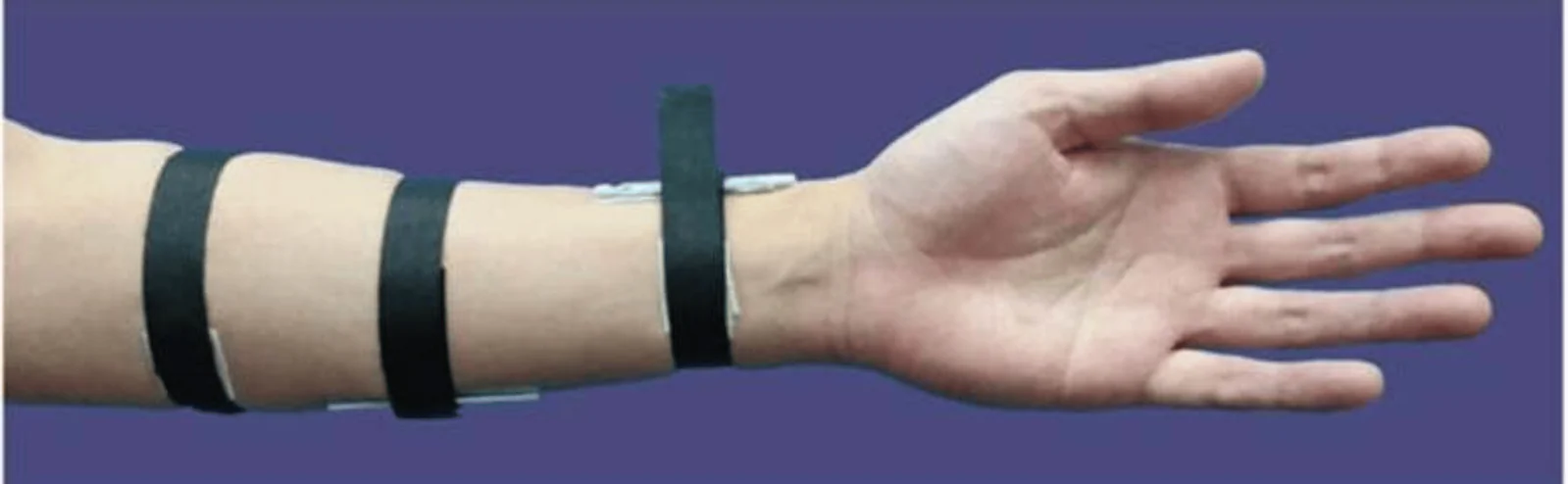
Placement of optical fiber force myography sensors on the forearm The optical fiber force myography transducers are secured at different positions around the forearm using straps to detect changes associated with hand postures. Image Credit: Fujiwara and Suzuki [22]; reproduced under the Creative Commons Attribution (CC BY) license (CC BY 4.0 Deed)

The OFS works on an entirely different principle. It is not a single component but an assembly comprising a light source, a light detector, an optical fiber, and an optomechanical transducer. The transducer compartment houses corrugated plates of predetermined geometry with an optical fiber threaded through them (Figure 4). The sensor is strapped over the muscle (Figure 5), with the light source launching light through the fiber to the detector. When the muscle contracts, it exerts force on the corrugated plates, which transfer that force as pressure onto the fiber. The resulting microbending deformation alters the optical properties of the fiber, including its transmitted light intensity, and that change is detected and measured. In both the FSR and OFS cases, a mechanical event is converted into a measurable electrical or optical output [22].

**Figure 6:**
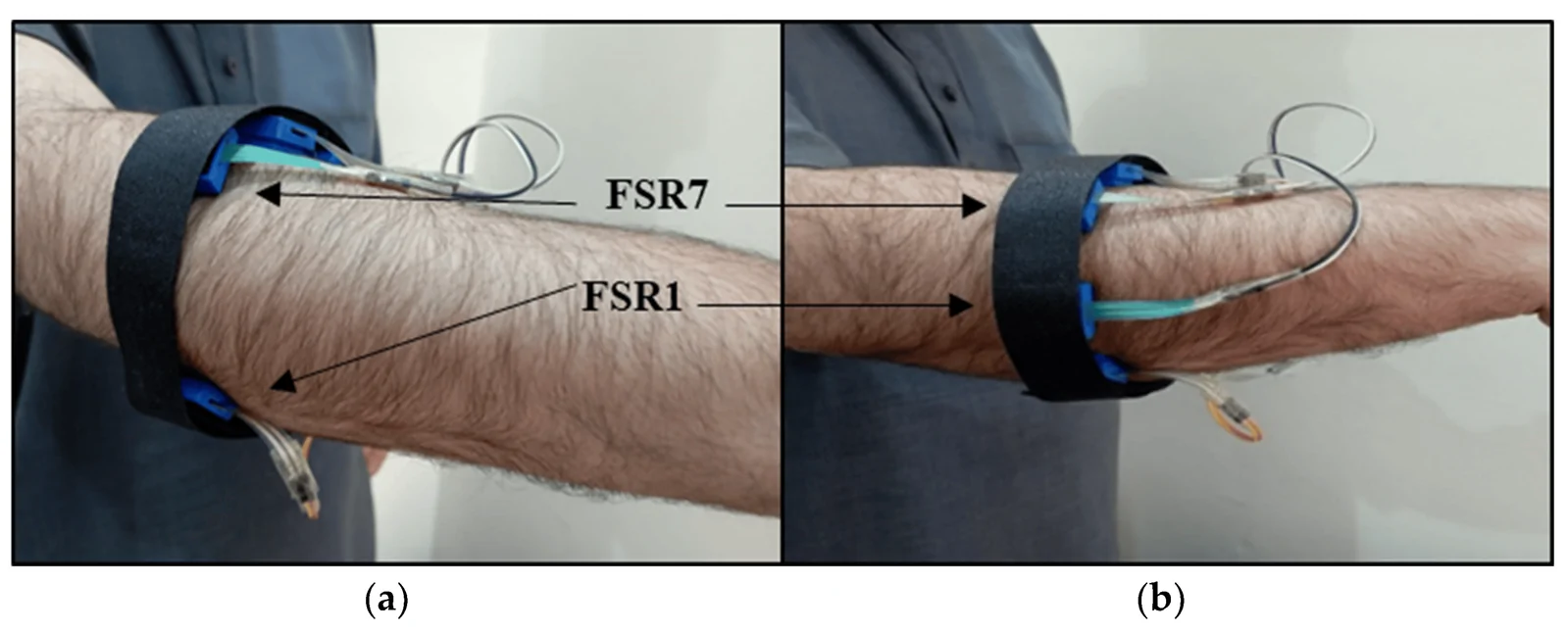
Force myography armband configurations on the forearm (a) Arrangement A, in which seven force-sensitive resistor sensors are positioned at a fixed spacing of 4.5 cm along the armband. (b) Arrangement B, in which the seven sensors are uniformly distributed around the circumference of the forearm. FSR1 and FSR7 indicate the first and seventh force-sensitive resistor sensors, respectively. Image Credit: Rehman et al. [23]; reproduced under the Creative Commons Attribution (CC BY) license (CC BY 4.0 Deed)

Figure 6 shows FMG armband configurations on the forearm.

Signal Propagation and Acquisition

For the FSR, the acquired signal is a change in voltage arising from the sensor's change in resistance. A voltage divider circuit is the most common way to extract this signal, valued for its simplicity and ease of setup, and the conditioned signal is then passed by wire to the processing stage.

For the OFS, the signal travels along the optical fiber as light. A light source, typically an LED, guides light to a photodetector that measures its intensity. As the muscle presses on the fiber, the characteristics of the transmitted light change, and the photodetector registers this change for onward processing. Because the optical path carries no electrical current at the sensing site, this mode of transfer is inherently resistant to electromagnetic interference [22].

Signal Processing and Noise Resistance

FMG signal processing is comparatively light. For the FSR, the voltage divider output is buffered by an operational amplifier in a unity gain configuration and digitized by an analogue to digital converter. Reported sampling rates for FMG generally fall in the tens to low hundreds of hertz; one detailed investigation suggests minimum sampling frequencies of roughly 54-84 Hz are sufficient for forearm and wrist signals depending on whether the movements are isometric or dynamic, with finger movements being the most demanding case [24]. Many FMG studies apply only minimal filtering, and some operate on raw signals, because the mechanical signal is relatively clean to begin with.

The processed signals are well suited as inputs to machine learning classifiers such as support vector machines and k-nearest neighbors for distinguishing hand gestures. A central advantage of FMG is its low susceptibility to noise: unlike EMG, which picks up electromagnetic interference from nearby electronic devices, FMG sensors register direct mechanical pressure and are largely indifferent to electrical noise. They are also less affected by the electrode displacement and skin condition problems that degrade EMG, since they do not rely on stable electrical contact. The principal sources of error are instead mechanical: sensor placement variability, externally applied pressure on the band, and anthropometric differences between users.

Real-Time Processing Capabilities

FMG is well suited to real-time control because of its relatively straightforward acquisition and limited preprocessing requirements. In the study by Fujiwara and Suzuki, an optical fiber FMG system achieved an average classification accuracy of 99.7% across nine hand postures under the study's specific experimental and validation conditions [22]. Li and colleagues reported greater than 99% within-session accuracy for the classification of 17 finger motions using a 32-sensor FSR array and a support vector machine classifier; approximately 79-98% accuracy was reported during intra-wearing cross-session testing when the array remained in place and was not removed or reset between sessions [25]. These results demonstrate the potential of FMG for gesture recognition but should not be interpreted as directly superior to accuracy values reported for other myography modalities because the available studies differ substantially in participant populations, numbers and types of gestures, sensing configurations, classifiers, and validation procedures.

Suitability for Prosthetic Control

FMG is a promising candidate for prosthetic hand control because its noninvasive, wearable configuration is well suited to gesture recognition and grip selection. Importantly, the evidence is no longer confined to able-bodied participants. In a feasibility study, Cho and colleagues predicted as many as 11 distinct grips in individuals with transradial amputation [26], and in a direct comparison, a prototyped FMG band classified hand gestures with accuracy exceeding that of concurrently recorded surface EMG [27]. The main limitation is depth: because FMG senses surface-level volumetric change, it is less able to resolve the activity of deep muscles than a technique such as SMG.

Relative Cost and System Complexity

FSR-based FMG has comparatively low hardware complexity because it can be implemented using FSRs, relatively simple conditioning circuitry, and conventional data acquisition hardware [19,21,22]. Optical fiber FMG requires more specialized sensing components, including a light source, an optical fiber, a photodetector, and an optomechanical transducer [19,22], but its downstream signal processing requirements can remain relatively straightforward. Neither approach requires implantation, which reduces procedural complexity compared with invasive sensing interfaces. Overall, FSR-based FMG represents one of the simpler hardware configurations among the modalities considered, while optical fiber FMG requires more specialized sensing hardware.

SMG

FMG and surface EMG both acquire information at or near the skin surface. SMG takes a fundamentally different route, looking inside the limb to image the muscles directly.

Overview

In the search for human-machine interfaces that offer more natural and reliable control than the established myographic methods, researchers have turned to SMG. Rather than sensing electrical activity or surface pressure, SMG uses ultrasound imaging to visualize muscle contraction directly, tracking the structural changes in muscle tissue as the limb moves. Its defining advantage is depth: because ultrasound penetrates the tissue, SMG resolves the activity of both superficial and deep muscles at high spatial resolution, capturing detailed anatomical information that surface techniques cannot reach [28]. This ability to spatially separate individual muscles, including those lying deep within the forearm, is what makes SMG attractive for fine-grained prosthetic control.

Principle of Operation

**Figure 7:**
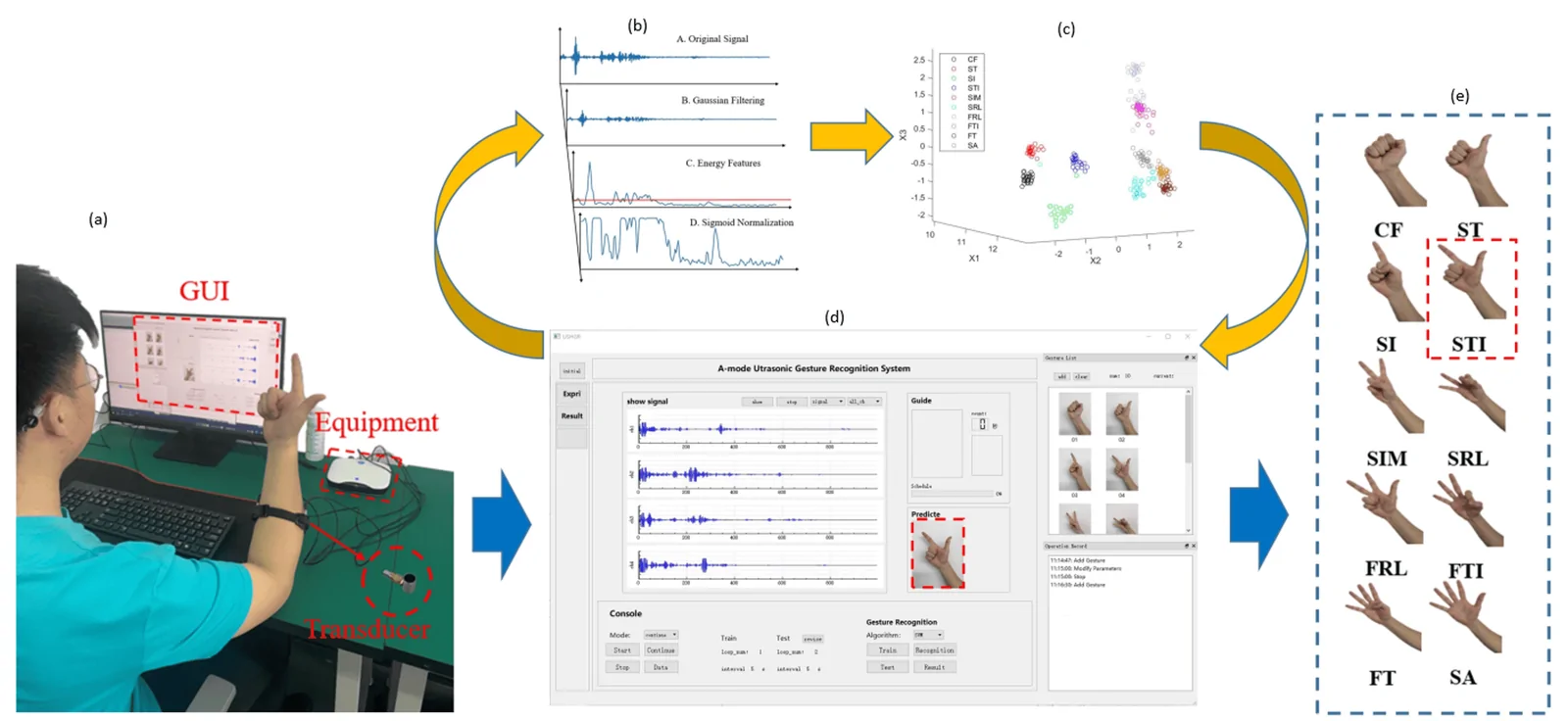
Framework of the wearable A-mode ultrasound-based real-time hand gesture recognition system (a) Experimental setup showing the wearable ultrasound transducer, acquisition equipment, and GUI used for real-time signal collection. (b) Signal processing workflow applied to acquired A-mode ultrasound signals, including filtering, feature extraction, and normalization. (c) Feature space visualization demonstrating the separation of different hand gesture classes. (d) User interface for signal visualization, gesture recognition, and system control. (e) Representative hand gestures used for classification: CF (closed fist), ST (stop sign), SI (single index), STI (single thumb-index), SIM (single middle finger), SRL (spread ring-little), FRL (flexed ring-little), FTI (flexed thumb-index), FT (flexed thumb), and SA (spread all). GUI: graphical user interface Image Credit: Lu et al. [31]; reproduced under the Creative Commons Attribution (CC BY) license (CC BY 4.0 Deed)

Depending on the acquisition mode, SMG may use one-dimensional A-mode echo profiles or two-dimensional B-mode images. Ortenzi and colleagues used B-mode ultrasound to capture transverse images of the forearm [29]. Earlier work by Castellini and colleagues demonstrated that ultrasound image features could be used to predict finger positions [30]. More recently, Lu and colleagues demonstrated real-time gesture recognition using wearable A-mode ultrasound [31]. Muscle contraction produces repeatable changes in these ultrasound signals or images, which can then be processed to infer intended hand movements. As the user attempts a movement, the muscles deform in a characteristic, repeatable way, and the resulting changes in the ultrasound image are read by a classification algorithm trained to associate particular deformation patterns with particular intended movements (Figure 7). In a representative implementation, Ortenzi and colleagues used a portable ultrasound scanner with a linear transducer to capture B-mode images of the transverse section of the forearm, holding the probe in place with an elastic band fixed to a custom plastic cradle in order to suppress motion artefacts during use [29]. The captured image stream is then read frame by frame by the classifier to predict the intended hand configuration. Earlier work had already established that ultrasound image features are linearly related to finger position, which is the basis on which SMG predicts hand posture [30].

Signal Propagation and Acquisition

SMG differs fundamentally from the other techniques in that it requires no electrodes, no embedded mechanical sensors, and no light source at the skin. The ultrasound transducer both transmits and receives, and the resulting ultrasound signals or image data are passed to a processing unit. Ultrasound provides depth-resolved structural information that can distinguish deformation patterns associated with different hand states. Depending on the acquisition mode, the output may consist of one-dimensional echo signals or two-dimensional image data. B-mode provides spatially rich image information, whereas A-mode can provide a more compact signal representation suitable for wearable implementations. The tradeoff is that image quality, and therefore classification accuracy, depends critically on the transducer maintaining a stable position and viewing angle against the limb; even small shifts in coupling or angle can substantially alter the acquired image [32]. Mechanical stabilization, such as a secured housing, is therefore used to keep the signal consistent.

Signal Processing and Noise Resistance

The processing approach depends on the ultrasound acquisition mode. B-mode SMG can use image-based features and computer vision methods, whereas A-mode implementations analyze one-dimensional echo signals and derived features [29,31]. Using this approach on three intact volunteers, Ortenzi and colleagues found that a linear discriminant analysis classifier trained on histogram of oriented gradients (HOG) features achieved around 80% accuracy in categorizing 10 hand postures and grasps, but only around 60% accuracy in distinguishing graded levels of grip force [29]. In that small study, classification performance was substantially lower for graded force than for discrete postures and grasps, illustrating that SMG performance can depend strongly on the task and outcome being estimated. In terms of robustness, SMG is largely immune to the electromagnetic interference that troubles EMG, and it does not suffer the electrode displacement or skin condition problems of surface electrical methods. Thus, SMG is comparatively resistant to electrical interference but remains vulnerable to mechanically induced changes in transducer coupling and position [32].

Real-Time Processing Capabilities

Real-time SMG control is feasible, but ultrasound acquisition and image or signal analysis generally impose greater computational requirements than simpler waveform-based sensing approaches. These requirements, together with the need for compact wearable ultrasound hardware, remain important considerations for prosthetic implementation [31,32]. Lu et al. demonstrated wearable A-mode ultrasound gesture recognition in 10 participants, reporting an offline accuracy of 96.92±1.92%, a real-time recognition accuracy of 83.8±6.9%, and an action completion rate of 96.0±3.6% [31]. Yin et al. subsequently integrated a compact four-transducer A-mode ultrasound system into a prosthetic hand socket and evaluated six gestures in 20 able-bodied participants. The mean online classification accuracy was 91.5±6.4% while wearing the physical prosthesis and 96.5±3.0% without it [33].

Suitability for Prosthetic Control

SMG offers important advantages for prosthetic control, including access to deformation of both superficial and deeper muscles and avoidance of the surface electrode interface limitations associated with surface EMG. Importantly, wearable ultrasound has also been evaluated directly in individuals with upper limb loss. Yang et al. studied four participants with transradial amputation performing six finger gestures together with wrist pronation/supination and demonstrated simultaneous prediction of hand and wrist motion, with comparative analyses favoring ultrasound over surface EMG under the study conditions [34]. Engdahl et al. subsequently demonstrated real-time control of a physical prosthetic hand by an individual with congenital bilateral limb absence, including completion of functional tasks across changing arm positions, three hours of use without classifier retraining, and simulated donning and doffing [35]. More recently, Nazari and Zheng developed a wireless wearable SMG interface for control of a 6-DOF prosthetic hand. After achieving 100% classification success for nine gestures in an offline study of 10 healthy participants, the system was evaluated in real time in two amputees performing functional hand tasks [36]. These findings strengthen the translational evidence for SMG, although available prosthesis user studies remain small. Practical barriers include maintaining consistent transducer coupling during use, integrating ultrasound hardware into a comfortable wearable system, and ensuring robust performance across changing arm positions and activities [32].

Relative Cost and System Complexity

SMG requires specialized ultrasound sensing hardware together with appropriate acoustic coupling, transducer stabilization, data acquisition, and signal or image processing resources [28-32]. These requirements give SMG comparatively high system complexity relative to simpler surface pressure- or camera-based approaches. Wearable A-mode implementations can reduce some of the acquisition and processing demands associated with conventional B-mode imaging, but integration of the transducer and associated electronics into a compact and stable prosthetic interface remains an important engineering challenge [31,32]. Consequently, the principal practical burden of SMG is better characterized in terms of specialized hardware and integration complexity than by a single monetary estimate.

OMG

The final technique returns to optical sensing, using light-based measurements rather than electrical or force signals to characterize contraction-related changes in the forearm.

Overview

OMG, first introduced in 2015, is an emerging noninvasive technique that monitors muscle activity using light, tracking the changes in light reflection, absorption, or scattering caused by muscle deformation during contraction [37]. It offers a promising alternative to the techniques discussed earlier. Two main approaches have emerged: camera-based OMG and near-infrared (NIR) photoelectric OMG.

Principle of Operation

**Figure 8:**
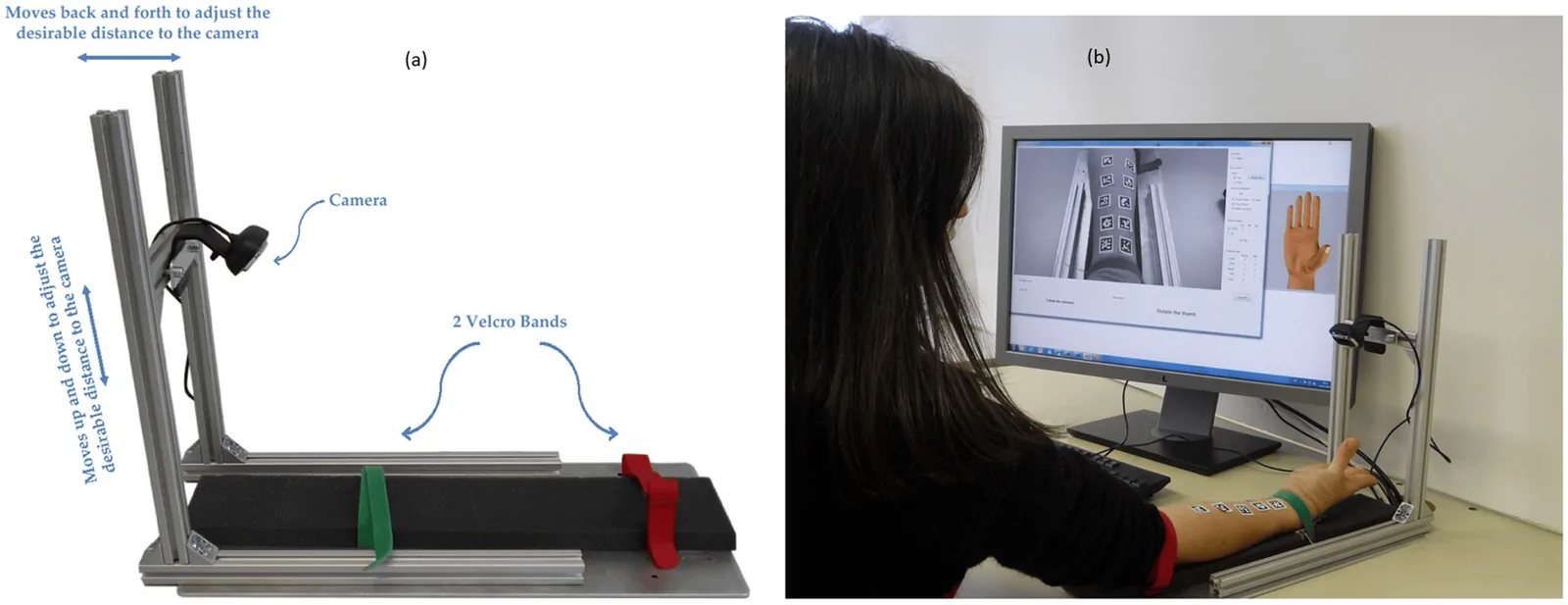
Experimental setup for optical myography-based finger movement detection (a) Adjustable experimental rig consisting of an off-the-shelf web camera and two Velcro bands used to stabilize the participant's forearm. The camera position can be adjusted vertically and horizontally to obtain the desired viewing distance and orientation. (b) Data acquisition setup showing the participant's forearm secured within the rig, with visual fiducial markers attached to the forearm, while the participant follows the hand movement stimulus displayed on the monitor. The camera tracks changes in the positions of the fiducial markers caused by forearm surface deformation and maps these changes to intended finger movements. Image Credit: Nissler et al. [38]; reproduced under the Creative Commons Attribution (CC BY) license (CC BY 4.0 Deed)

Camera-based OMG: Advances in computer vision have driven the development of camera-based OMG. A notable study by Nissler and colleagues demonstrated the feasibility of the approach. The setup used a standard web camera connected to a computer to observe the forearm, while visual fiducial markers placed on the skin tracked the surface deformations produced by muscle contraction. Using data from 10 intact participants, the system was trained to map forearm surface deformation to four movement dimensions: thumb rotation, thumb flexion, index finger flexion, and combined flexion of the middle, ring, and little fingers (Figure 8) [38]. The method reported low normalized error for finger flexion and higher error for thumb rotation, the latter attributed to the deeper location of the thumb-related muscles, which are less visible at the forearm surface than the superficial flexor muscles responsible for finger flexion. The fixed camera-to-forearm configuration, however, limits the practicality of this approach for mobile prosthetic use [38].

**Figure 9:**
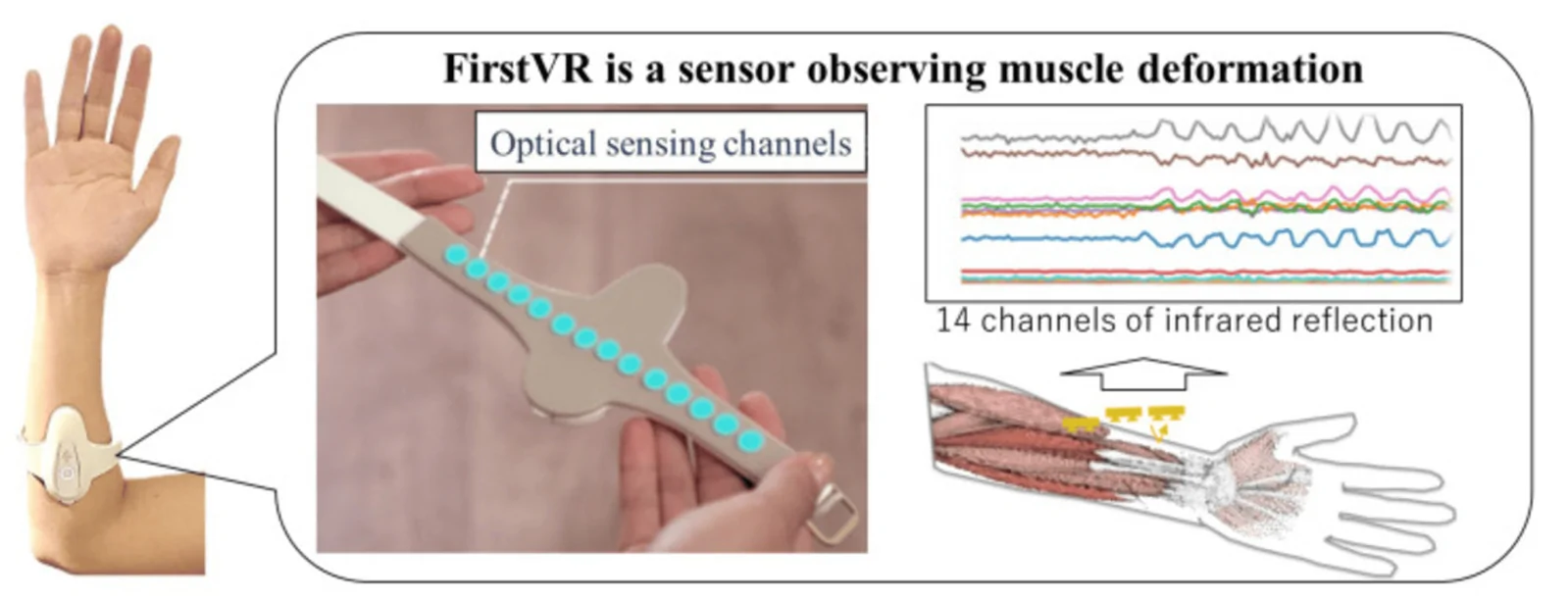
Near-infrared optical myography sensor used for forearm muscle deformation sensing The FirstVR wearable sensor incorporates 14 near-infrared optical myography channels for detecting changes in forearm muscle deformation during hand-gripping tasks. Image Credit: Miyake et al. [39]; reproduced under the Creative Commons Attribution (CC BY) license (CC BY 4.0 Deed)

NIR photoelectric OMG: To address the mobility and lighting limitations of the camera-based approach, researchers have explored NIR photoelectric OMG. The study by Miyake and colleagues used a band-type sensor, a 14-channel wearable device based on NIR optical sensing, to monitor forearm skeletal muscle activity [39]. The principle is based on how NIR light interacts with tissue: The FirstVR emits NIR light into the underlying tissue and detects the returned optical signal. During muscle contraction, deformation of the skeletal muscle and overlying skin alters the signal measured by the sensor channels (Figure 9) [39]. In that study, 14 healthy participants performed gripping tasks including squeezing a hand gripper, a ball, and a paper balloon, clenching the fist, and crumpling paper. The system successfully detected muscle deformation features across these tasks, and its time-series measures captured both the variability and the periodicity of the applied load [39].

Signal Propagation and Acquisition

In the camera-based method, the markers on the skin track muscular motion, the camera captures that motion visually, and the data are transferred to a computer. For prosthetic applications, a wearable band carrying multiple emitter and detector pairs maps muscle activity across the forearm: the light penetrates the skin, interacts with the underlying muscle tissue, and returns to the detector, where the change in the received signal encodes the contraction.

Signal Processing and Noise Resistance

The camera-based method removes the direct current and high-frequency components of the tracked marker signals using a second-order Butterworth band-pass filter before mapping the marker trajectories to intended finger motions [38]. Because the method is optical, it is not affected by electrical interference; however, performance may be compromised by image blur, marker occlusion, and uncorrelated movement of the forearm relative to the camera. Changes in brightness and contrast had comparatively little effect in the reported experimental setup [38]. NIR OMG is similarly resistant to electromagnetic interference and skin impedance effects but remains susceptible to ambient light contamination, sensor lift-off, skin pigmentation, scarring, and moisture [40]. Thus, optical sensing offers strong resistance to electrical noise but remains vulnerable to mechanical and optical disturbances.

Real-Time Processing Capabilities

Camera-based OMG has demonstrated finger movement estimation using marker-based optical data, but the cited study primarily evaluated offline processing rather than a complete wearable real-time prosthetic control loop [38]. NIR-based OMG has demonstrated wearable time-series monitoring of forearm muscle deformation during gripping tasks [39]. More recently, Khalikov et al. demonstrated continuous real-time decoding using a wearable 50-channel OMG wristband. Eight able-bodied participants and one participant with limb loss used the interface for continuous cursor control and gaming tasks following gesture-based calibration [41]. This represents an important advance in real-time wearable OMG control, although direct long-term control of a physical hand prosthesis in a larger population of prosthesis users remains to be established.

Suitability for Prosthetic Control

Camera-based OMG has demonstrated finger movement estimation under controlled conditions, but the requirement for a stable camera-to-forearm arrangement restricts wearable application [37,38]. NIR-based OMG is more compatible with wearable sensing, and recent work has extended the approach from muscle deformation monitoring to continuous real-time human-machine control [39-41]. Nevertheless, evidence from individuals with limb loss remains limited, and larger studies involving direct physical prosthesis control, long-term use, sensor repositioning, and activities of daily living are still needed.

Relative Cost and System Complexity

The system complexity of OMG depends strongly on the sensing approach. Camera-based OMG can be implemented using conventional imaging hardware and fiducial markers, giving it relatively low prototype hardware complexity [37,38]; however, maintaining a stable camera-to-forearm geometry complicates practical integration into a mobile prosthetic system. NIR-based OMG requires multiple optical emitters and detectors together with associated acquisition electronics, resulting in greater sensing hardware complexity than a basic camera-based configuration [39,41]. Wearable implementation is feasible, but the technology remains less mature for prosthetic control than established surface EMG or FMG systems. Accordingly, relative implementation complexity, rather than a single monetary estimate, provides a more defensible basis for comparison between the OMG variants.

Machine learning and signal processing

Each of the techniques discussed so far produces a raw signal that must be transformed before it can drive a prosthesis, and across all of them, this transformation follows a broadly similar path. Because the quality of that path determines how accurately a user's intention is recognized, machine learning and signal processing have become central to modern myographic control, and they are the common thread that links the four modalities.

Myographic signals, surface EMG in particular, are inherently noisy and non-stationary, varying both within and between recording sessions, so they require conditioning before use [14]. Preprocessing typically begins with filtering to suppress noise, followed by normalization and by segmentation of the continuous signal into short analysis windows. This windowing is what allows the system to issue control decisions at a steady rate while keeping latency low enough for responsive control.

Feature extraction then reduces each window to a compact set of descriptors that capture the information relevant to movement intention. Time domain features such as mean absolute value and waveform length are popular because they are computationally cheap and effective, which suits them to real-time use, while frequency domain features add complementary information about the spectral content of muscle activity. More elaborate time-frequency representations can improve the richness of the data at the cost of additional computation, and the careful selection of a feature set has been shown to materially affect classification performance [42].

The extracted features are passed to a classifier that maps them to an intended gesture. Traditional methods such as linear discriminant analysis and support vector machines remain widely used because they are fast, robust, and well suited to embedded, real-time control [43]. Their main weakness is that they depend on manually engineered features and tend to degrade when signal conditions change, for example, through muscle fatigue, sweating, or electrode displacement. To overcome this, deep learning approaches, especially convolutional neural networks, have been explored; these can learn discriminative features directly from the raw or lightly processed signal and can improve classification accuracy, particularly across subjects and sessions [44]. The tradeoff is their appetite for computation and large training datasets, which complicates deployment in compact, power-constrained prosthetic hardware.

Longitudinal signal variability remains an important challenge for myography-based classifiers. Adaptive and online learning approaches seek to address this problem by updating the model as signal characteristics change over time [45].

Comparative analysis

Having examined each technique individually, the four modalities selected for detailed comparison, that is, EMG, FMG, SMG, and OMG, were compared across the six predefined domains of signal propagation, signal accuracy, noise resistance, real-time processing, suitability for prosthetic control, and relative cost and system complexity.

Because the underlying studies differed substantially in participant populations, amputation status, sample size, sensing hardware, number and type of gestures, classification algorithms, and validation procedures, reported performance values were not treated as directly equivalent or mathematically pooled. The comparison therefore represents a narrative, criterion-by-criterion synthesis of the available evidence rather than a quantitative ranking of the modalities. No weighting scheme was applied because the manuscript did not prespecify stakeholder-derived weights for the six criteria and assigning such weights retrospectively would introduce additional subjective assumptions rather than resolve the heterogeneity of the underlying evidence.

The selected modalities show distinct and application-dependent tradeoffs across the six predefined criteria (Table 1). Surface EMG benefits from clinical maturity and real-time capability but remains affected by recording variability and reduced stability across sessions [10,13,14]. FMG provides relatively straightforward mechanical sensing and resistance to electromagnetic interference, with high classification performance reported in several controlled studies [19,22,25-27]. SMG provides information from both superficial and deeper muscle structures and has now been demonstrated in wearable online prosthetic control configurations and in individuals with transradial amputation, although transducer stability, wearable integration, and signal or image processing requirements remain important considerations [28-34]. OMG avoids electrical sensing altogether; camera-based implementations remain constrained by camera-to-forearm geometry, whereas NIR-based systems are more compatible with wearable configurations, and recent work has demonstrated continuous real-time OMG decoding, although the direct prosthetic user evidence base remains comparatively small [37-41].

**Table 1:** Qualitative comparison of selected myography techniques for prosthetic hand control Six predefined criteria were retained for the comparison: signal propagation, signal accuracy, noise resistance, real-time processing, suitability, and relative cost and system complexity. An additional evidence context row was included as an interpretive note, rather than as a seventh performance criterion, to identify important differences in participant populations, amputation status, sample sizes, sensing configurations, gesture sets, classification algorithms, and validation procedures. Because the underlying studies are methodologically heterogeneous, reported performance values are presented within their individual study contexts and should not be interpreted as direct head-to-head comparisons. No numerical scores, weighting scheme, total scores, or aggregate ranking were applied because the available evidence does not provide a validated basis for quantitatively ranking the modalities. EMG: electromyography; FMG: force myography; FSR: force-sensitive resistor; SMG: sonomyography; NIR: near-infrared; OMG: optical myography

Criteria	Surface EMG	Intramuscular EMG	Optical fiber FMG	FSR-based FMG	SMG	NIR-based OMG	Camera-based OMG
Signal propagation	Detects electrical activity primarily from superficial muscles. Recorded signal characteristics are influenced by muscle depth, overlying tissue, and electrode placement [10,11,15-17].	Records localized electrical activity from within target muscles, providing greater selectivity than surface recording but requiring invasive electrode placement [46].	Detects mechanical muscle deformation through pressure-induced microbending of an optical fiber, with the resulting signal transmitted optically [19,21,22].	Detects surface pressure and volumetric changes produced by muscle contraction using FSRs [19,21,23-27].	Uses ultrasound to detect structural deformation of superficial and deep muscles, providing access to deeper muscular activity than surface-based methods [28-32].	Uses NIR optical sensing to detect changes associated with forearm muscle deformation during contraction [39,41].	Detects visible forearm surface deformation using a camera and fiducial markers; deformation generated by deeper muscles is less readily observed at the surface [37,38].
Signal accuracy	High within-session classification performance has been reported, but performance may deteriorate across days when classifiers are not recalibrated [13,14].	High and sustained control performance has been demonstrated with implanted EMG interfaces, although results depend on implantation strategy, target muscles, task, and decoding protocol [46].	Fujiwara and Suzuki reported 99.7% average accuracy across nine hand postures in six participants under their specific experimental and validation conditions [22].	Li et al. reported >99% within-session accuracy for 17 finger motions, with approximately 79-98% accuracy in intra-wearing cross-session testing when the FSR array was not removed or reset [25]. Performance varies across other FMG studies according to task and protocol [23,26,27].	Performance varies substantially by task and acquisition method. Ortenzi et al. reported approximately 80% accuracy for the classification of hand postures/grasps and approximately 60% when classifying different grip force levels in three intact participants [29]. Other ultrasound studies have reported different performance under different experimental conditions [28,30,31].	Feasibility for detecting changes in forearm muscle activity during different gripping tasks has been demonstrated, but directly comparable prosthetic gesture classification accuracy remains limited [39,41].	Finger movement estimation has been demonstrated experimentally, with normalized root mean square errors reported rather than classification accuracies directly comparable with the other modalities [37,38].
Noise resistance	Susceptible to electromagnetic interference, motion artefacts, changes in skin impedance, sweating, electrode displacement, and physiological variability [10,11,15,16].	Avoids several skin electrode interface problems associated with surface EMG, although signal characteristics can still vary between recording sessions and depend on implanted electrode stability [46].	Resistant to electromagnetic interference because sensing and transmission are optical; measurements can nevertheless be influenced by transducer placement and applied mechanical loading [19,22].	Not directly susceptible to electromagnetic interference, but measurements may be affected by sensor placement, band pressure, externally applied force, and changes in limb sensor geometry [19,23-27].	Largely resistant to electromagnetic interference but sensitive to transducer movement, position, orientation, acoustic coupling, and mechanical loading [28-32].	Avoids electrical interference and skin impedance problems associated with EMG, although optical measurements can be affected by optical and placement-related disturbances [39,41].	Resistant to electrical interference but susceptible to motion blur, marker occlusion, and relative movement between the camera and forearm. The reported experimental system showed relative robustness to controlled changes in brightness and contrast [38].
Real-time processing	High temporal resolution supports real-time prosthetic control, although preprocessing, feature extraction, and classification add computational requirements [10,17,43].	Real-time prosthetic control has been demonstrated using implanted EMG interfaces, including sustained long-term performance [46].	The relatively simple optical intensity acquisition and classification pipeline is compatible with rapid processing; however, the cited 99.7% study primarily evaluated posture classification performance rather than a complete real-time prosthetic control loop [22].	Real-time and online gesture control applications are feasible using comparatively simple signal acquisition and conventional classifiers [23,26,27].	Real-time recognition has been demonstrated with wearable A-mode ultrasound. Lu et al. reported 83.8%±6.9% real-time recognition accuracy for 10 gestures [31]. Yin et al. subsequently integrated a four-transducer A-mode ultrasound system into a prosthetic hand socket and reported mean online classification accuracies of 91.5% while wearing the physical prosthesis and 96.5% without it [33].	Wearable time-series monitoring of muscle deformation has been demonstrated [39], and continuous real-time OMG decoding has subsequently been demonstrated using a 50-channel wearable wristband in eight able-bodied participants and one participant with limb loss [41]. Direct long-term physical prosthetic hand control remains insufficiently established.	The cited camera-based OMG study primarily used offline marker processing rather than demonstrating a complete real-time wearable prosthetic control system [38].
Suitability	Noninvasive, clinically established, and widely used in commercial upper limb prostheses. Limitations include electrode displacement, signal variability, recalibration requirements, and difficulty maintaining stable multidimensional control [8,10,17,18].	Can provide selective and durable control signals, but electrode implantation requires an invasive procedure, limiting its applicability to users unwilling or unable to undergo surgery [46].	Promising for wearable gesture recognition because of electromagnetic interference resistance, relatively simple signal acquisition, and strong classification performance in controlled studies; however, long-term evidence in prosthesis users remains limited [19,22,47].	Wearable and comparatively simple and has been investigated for gesture and grip recognition, including feasibility testing in individuals with transradial amputation [19,23-27]. Performance remains dependent on consistent sensor limb interaction.	Offers access to both superficial and deeper muscular deformation and has substantial potential for dexterous prosthetic control. Wearable ultrasound has been investigated in individuals with transradial amputation for the simultaneous prediction of finger gestures and wrist rotation [34], although transducer stability, wearable integration, and processing requirements remain barriers to routine everyday use [28-34].	Provides a noninvasive wearable approach to monitoring forearm muscle deformation, but direct validation for long-term prosthetic hand control in individuals with amputation remains limited [39,41].	Fully noninvasive and uses inexpensive conventional imaging hardware, but the need for a stable camera-to-forearm relationship and the current reliance on controlled experimental arrangements limit mobile prosthetic use [37,38].
Relative cost and system complexity	Requires surface electrodes, amplification, signal conditioning, filtering, classification hardware, and periodic calibration. Hardware is clinically mature, but signal processing requirements contribute to system complexity [10,16].	Has greater procedural and system complexity because electrodes must be implanted or inserted into muscle and may require surgical intervention and specialized follow-up [46].	Requires an optical source, optical fiber, photodetector, and optomechanical transducer. The sensing hardware is specialized, although signal acquisition and downstream processing can be relatively simple [19,22].	Uses relatively simple FSRs, conditioning circuitry, and data acquisition hardware, giving it comparatively low hardware complexity [19,21,23].	Requires ultrasound transducers, appropriate acoustic coupling, mechanical stabilization, and signal/image processing hardware, resulting in comparatively high system complexity [28-32].	Requires multiple optical emitters and detectors together with associated acquisition electronics. Wearable implementation is feasible, but the technology is less mature for prosthetic control than EMG or FMG [39,41].	Can use conventional cameras and relatively simple fiducial markers, giving low prototype hardware complexity; however, maintaining the required camera-to-forearm geometry complicates practical prosthetic integration [37,38].
Evidence context (interpretive note; not an additional scoring criterion)	Evidence includes both able-bodied participants and individuals with transradial amputation. Longitudinal studies demonstrate that performance measured within one session should not automatically be generalized to performance across days [13,14].	Long-term clinical feasibility has been demonstrated, including implanted EMG from regenerative peripheral nerve interfaces and residual muscles, but available studies involve small numbers of individuals and invasive procedures [46].	The frequently cited 99.7% accuracy came from six participants performing nine postures using three optical fiber transducers and study-specific artificial neural network classification. It should not be interpreted as directly superior to values obtained using different tasks, populations, or validation methods [22].	The >99% result was obtained for 17 finger motions during in-session validation. The reported 79-98% cross-session results were obtained without removing/resetting the FSR array; therefore, these values should not be interpreted as performance after complete sensor repositioning [25]. Other FMG studies differ in sensor configuration, participant population, and gesture set [23,26,27].	The evidence includes studies using different ultrasound modes, tasks, classifiers, and participant groups. For example, the approximately 80% posture/grasp and 60% grip force results reported by Ortenzi et al. were obtained in three intact participants using B-mode ultrasound, whereas Lu et al. evaluated real-time recognition using wearable A-mode ultrasound under a different protocol [29,31].	Miyake et al. evaluated 14 healthy participants performing gripping tasks and demonstrated wearable muscle deformation monitoring [39]. Khalikov et al. subsequently evaluated continuous real-time OMG control in eight able-bodied participants and one participant with limb loss [41]. The latter study strengthens the evidence for real-time wearable OMG but does not yet constitute large-scale long-term validation of physical prosthetic hand control.	The cited studies involved 10 intact participants using a fixed camera, a stabilized forearm, and fiducial markers. Performance was reported using normalized root mean square error rather than classification accuracy directly comparable with the other modalities [37,38].

The methodological appraisal reinforces the need to interpret the comparative findings cautiously. Measurement procedures and experimental protocols were generally sufficiently described to support the interpretation of study-specific outcomes, but the evidence base was frequently limited by small samples, controlled laboratory tasks, and the predominance of able-bodied participants in several FMG, SMG, and OMG studies. Evidence obtained directly from individuals with upper limb amputation was available for selected EMG, FMG, and SMG investigations, for long-term implanted EMG control, and in a recent OMG investigation that included one participant with limb loss; however, these studies generally involved substantially smaller participant samples than studies conducted exclusively in able-bodied populations.

Discussion and future directions

The comparative evidence indicates that modality selection for prosthetic hand control is inherently application-dependent. The major practical challenge is therefore not identifying a universally superior sensor, but balancing signal robustness, real-time responsiveness, wearability, sensing depth, invasiveness, and system complexity for a particular user and use case. This also provides a rationale for investigating hybrid systems that combine complementary sensing modalities.

Beyond muscle-based sensing, peripheral nerve interfaces represent a more direct approach to decoding motor intent and potentially restoring sensory information. Electroneurographic and other nerve-based interfaces aim to interact with neural signals closer to their source rather than infer intention from residual muscle activity [48]. These approaches remain comparatively experimental and involve different surgical and translational considerations from the noninvasive modalities reviewed here.

A particularly important development concerns the long-term signal stability that has challenged surface methods. Work on regenerative peripheral nerve interfaces (RPNIs) combined with implanted EMG electrodes has demonstrated durable signal quality and long-term prosthetic control. Vu et al. reported that RPNI signal-to-noise ratio remained greater than 15 for up to 276 days in one participant and 1,054 days in another. The latter participant maintained real-time prosthetic control performance above 94% for 604 days without recalibration and completed a multi-grasp coffee-making task with 99% accuracy for 611 days without recalibration [46]. These findings suggest a potential route past the repeated recalibration that limits conventional surface myoelectric control, although the approach requires surgery and the study involved only two participants.

In parallel, artificial intelligence is reshaping how prosthetic control is conceived. Rather than simply classifying gestures, AI-driven systems can learn and adapt to an individual user, personalizing control to feel more intuitive and helping to reduce latency and cognitive load [49]. The longer-term ambition is bidirectional, closed-loop control, in which the prosthesis not only receives motor commands but also returns sensory information to the user. Sensory feedback strategies are therefore an important component of efforts to make prosthetic interaction more natural and functionally useful [12].

Advances in wearable sensor technology are making these ambitions more practical. Flexible, skin-conformal electronics have shown that high-quality physiological signals can be acquired from unobtrusive, comfortable devices [50], and prosthetic systems are beginning to exploit this. One recent pilot study developed a hand controlled not by myoelectricity but by wireless wearable sensors, which extends usable control to individuals with very short residual limbs or paralysis for whom conventional myoelectric sensing is unsuitable while reducing fatigue compared with a conventional myoelectric hand [51]. Such work points toward prostheses that are lighter, more adaptable, and accessible to a broader range of users.

Beyond the four modalities selected for detailed comparison, other muscle-sensing approaches are also relevant to prosthetic control. Mechanomyography detects the low-frequency mechanical vibrations produced during muscle contraction and has been investigated as a noninvasive human-machine interface; however, its contemporary prosthetic control evidence base remains smaller than that of surface EMG and FMG [40]. Emerging magnet-based approaches provide another direction by tracking implanted magnetic markers associated with residual muscle movement. Early myokinetic and related magnetic-tracking systems have demonstrated the feasibility of deriving prosthetic control signals from individual muscle movements, although these approaches require implanted markers and currently have a substantially smaller clinical evidence base than established surface techniques [52,53]. These modalities were therefore acknowledged as emerging complementary approaches rather than incorporated into the primary four-modality comparison.

Taken together, these directions suggest that the next generation of prosthetic hand interfaces will be hybrid and adaptive rather than reliant on a single sensing principle, blending the practical strengths of techniques such as FMG with the precision of optical and neural interfaces, the intelligence of adaptive machine learning, and the restored sensation of closed-loop feedback. Realizing this will require continued progress on signal stability, computational efficiency, sensor comfort, and clinical translation, but the trajectory of the field gives good reason for optimism.

Future comparative studies could incorporate formal multi-criteria decision frameworks in which criterion weights are derived prospectively from prosthesis users, clinicians, prosthetists, and engineers rather than assigned retrospectively by review authors.

## Conclusions

Myography provides several viable approaches for translating residual muscle activity into control signals for upper limb prostheses, but the available modalities differ substantially in sensing principle, robustness, processing requirements, invasiveness, wearability, and maturity of clinical evidence. Surface EMG remains the most clinically established approach and is widely used in commercial prosthetic systems, although its performance can be affected by electrode displacement, skin-related factors, noise, and reduced classification stability across recording sessions. FMG offers a comparatively simple mechanical sensing approach that is resistant to electromagnetic interference, and both FSR-based and optical fiber implementations have demonstrated strong gesture classification performance in controlled studies. SMG provides access to deformation of both superficial and deeper muscles and has demonstrated real-time and functional prosthetic control feasibility, including evaluation in individuals with upper limb loss. However, stable transducer positioning, wearable integration, acoustic coupling, and comparatively greater acquisition and processing requirements remain important practical limitations. OMG provides a fully noninvasive optical sensing approach, with NIR-based systems showing promise for wearable applications, although evidence for sustained prosthetic hand control remains comparatively limited.

Direct numerical ranking of these modalities is not justified by the current literature because the available studies differ substantially in participant populations, amputation status, gesture sets, sensing configurations, classification algorithms, and validation procedures. Optical fiber FMG is nevertheless a promising option for portable prosthetic hand control because controlled studies have reported high gesture classification performance together with resistance to electromagnetic interference and relatively straightforward signal processing. This should not be interpreted as evidence that optical fiber FMG is universally superior to EMG, SMG, or OMG. Instead, modality selection should depend on the requirements of the intended application, including robustness, sensing depth, invasiveness, real-time performance, wearability, long-term stability, and system complexity.

Future research should emphasize longitudinal evaluation in individuals with upper limb amputation, including performance across repeated donning and doffing, daily activities, and changing physiological conditions. Hybrid sensing approaches that combine complementary modalities, such as FMG with EMG or optical sensing, may also improve robustness by reducing reliance on a single signal source. Advances in adaptive machine learning, wearable sensor design, and closed-loop sensory feedback may further improve the reliability and usability of future prosthetic hand control systems.

## Appendices

Appendix A

**Table 2:** Database-specific search strategies used for the narrative review literature search The final structured searches and record exports were completed on August 17, 2026. PubMed searches were restricted to Title/Abstract fields and to publications dated January 1, 2000, through August 16, 2026. IEEE Xplore searches used the corresponding Boolean combinations of modality and application terms in All Metadata fields, with publication years limited to 2000-2026. Where necessary, large IEEE Xplore result sets were exported in non-overlapping publication year blocks without altering the underlying search terms or eligibility criteria. Because the IEEE Xplore Advanced Search interface did not reliably execute the nested SMG Boolean expression as a single fielded query, the SMG strategy was implemented as two component searches, which were subsequently combined and deduplicated. English-language peer-reviewed journal articles and full conference papers were considered during relevance screening rather than through a database-level language filter. Earlier seminal publications predating 2000 were retained when they provided important historical or methodological context. The additional search for mechanomyography and magnet-based muscle tracking interfaces was conducted to identify relevant emerging approaches outside the four modalities selected for detailed comparison.

Database	Search topic	Exact search strategy	Limits/settings
PubMed	Electromyography (EMG)	(("electromyography"[tiab] OR "surface electromyography"[tiab] OR "intramuscular electromyography"[tiab] OR myoelectric[tiab]) AND (prosthe*[tiab] OR "upper limb"[tiab] OR hand[tiab] OR finger*[tiab] OR wrist[tiab] OR forearm[tiab] OR "gesture recognition"[tiab] OR "human-machine interface"[tiab] OR "human machine interface"[tiab])) AND 2000/01/01:2026/08/16[dp]	Title/Abstract fields; publication dates January 1, 2000-August 16, 2026. English-language and publication-type eligibility assessed during screening
PubMed	Force myography (FMG)	(("force myography"[tiab] OR "force myographic"[tiab] OR "force myogram"[tiab] OR "force-sensitive resistor"[tiab] OR "force sensing resistor"[tiab]) AND (prosthe*[tiab] OR "upper limb"[tiab] OR hand[tiab] OR finger*[tiab] OR grip*[tiab] OR wrist[tiab] OR forearm[tiab] OR "gesture recognition"[tiab] OR "human-machine interface"[tiab] OR "human machine interface"[tiab])) AND 2000/01/01:2026/08/16[dp]	Title/Abstract fields; publication dates January 1, 2000-August 16, 2026. English-language and publication-type eligibility assessed during screening
PubMed	Sonomyography (SMG)	((sonomyography[tiab] OR (ultrasound[tiab] AND (muscle[tiab] OR forearm[tiab]))) AND (prosthe*[tiab] OR "upper limb"[tiab] OR hand[tiab] OR finger*[tiab] OR grip*[tiab] OR gesture*[tiab] OR wrist[tiab] OR "human-machine interface"[tiab] OR "human machine interface"[tiab])) AND 2000/01/01:2026/08/16[dp]	Title/Abstract fields; publication dates January 1, 2000-August 16, 2026. English-language and publication-type eligibility assessed during screening
PubMed	Optical myography (OMG)	(("optical myography"[tiab] OR optomyography[tiab] OR "near-infrared optical myography"[tiab] OR "near infrared optical myography"[tiab]) AND (prosthe*[tiab] OR amput*[tiab] OR "upper limb"[tiab] OR hand[tiab] OR finger*[tiab] OR grip*[tiab] OR forearm[tiab] OR gesture*[tiab] OR "human-machine interface"[tiab] OR "human machine interface"[tiab])) AND 2000/01/01:2026/08/16[dp]	Title/Abstract fields; publication dates January 1, 2000-August 16, 2026. English-language and publication-type eligibility assessed during screening

Appendix B

**Table 3:** Structured eligibility framework for the narrative review The PICOS framework was modified to accommodate the technology-focused scope and heterogeneous evidence base of this narrative review. A formal comparator was not required because eligible evidence included single-modality feasibility, classification, and technical studies in addition to direct comparative studies. PICOS: Population, Intervention/Concept, Comparator, Outcomes, and Study design: EMG: electromyography; FMG: force myography; SMG: sonomyography; OMG: optical myography; MMG: mechanomyography

PICOS element	Inclusion criteria	Exclusion criteria
Population (P)	Human participant studies involving individuals with upper limb amputation or able-bodied participants when the study evaluated muscle-sensing approaches relevant to hand, wrist, forearm, or upper limb prosthetic/human-machine interface control. Technical studies without a clinical participant population could also be retained when they provided directly relevant information on sensing principles or system implementation.	Studies concerned exclusively with lower limb applications without transferable relevance to upper limb myographic control.
Intervention/Concept (I)	EMG, FMG, SMG, and OMG systems used to acquire muscle-related signals for gesture recognition, movement intention decoding, prosthetic control, or closely related human-machine interfaces. MMG and magnet-based muscle tracking approaches were included as supplementary emerging technologies for contextual discussion.	Purely mechanical prosthetic designs without a myographic or muscle-sensing control interface; muscle assessment techniques used solely for diagnostic purposes without relevance to prosthetic or human-machine control.
Comparator (C)	A formal comparator was not required. Eligible studies included single-modality evaluations, comparisons between sensing configurations or algorithms, and direct comparisons between modalities where available.	Studies were not excluded solely because they lacked a separate comparator or control modality.
Outcomes (O)	Evidence relevant to one or more predefined comparison domains: signal propagation, signal accuracy or movement classification performance, noise resistance/robustness, real-time processing, suitability for prosthetic control, and relative cost or system complexity. Additional relevant outcomes included wearability, cross-session stability, prosthesis user feasibility, and implementation limitations.	Studies that did not provide information relevant to the sensing characteristics, control performance, implementation, or practical limitations of myographic/prosthetic control systems.
Study design (S)	English-language peer-reviewed journal articles and full conference papers. Primary experimental studies were prioritized for modality-specific evidence; review articles were retained for technical context and citation chaining. Seminal publications predating 2000 were retained when necessary for important historical or methodological context.	Publications lacking sufficient relevance to the review objectives or addressing only unrelated prosthetic/mechanical topics.
Publication period	Primary structured search: January 1, 2000-August 16, 2026 for PubMed; IEEE Xplore searches were limited to publication years 2000-2026. Final searches and record exports were completed on August 17, 2026. Foundational pre-2000 publications could be retained when justified by historical or methodological importance.	Publications outside the primary date range that did not provide necessary foundational context.

Appendix C

**Table 4:** Search and screening flow for the updated narrative review literature search The counts describe the structured search and title/abstract screening process used to support this narrative review. Because the review was not conducted as a systematic review, records retained after screening were not advanced through a separately enumerated PRISMA-style full-text eligibility stage. Instead, retained records formed the evidence pool for targeted full-text evaluation and citation chaining, with primary studies providing direct evidence for the predefined comparison domains prioritized for detailed synthesis. Duplicate or overlapping occurrences reflect records retrieved through more than one database or modality-specific search and should not be interpreted solely as duplicate publications.

Stage	Number of records	Description
Records retrieved from PubMed	8,911	Combined search occurrences across the modality-specific PubMed searches
Records retrieved from IEEE Xplore	7,866	Combined search occurrences across the modality-specific IEEE Xplore searches
Total search occurrences before consolidation	16,777	Includes records retrieved by more than one modality-specific search
Duplicate or overlapping search occurrences consolidated	462	Cross-database and/or cross-query overlap
Unique records after consolidation	16,315	Master dataset used for title/abstract screening
Records excluded during screening	12,986	Excluded during objective eligibility or title/abstract relevance assessment
Records retained for further consideration	3,329	Evidence pool retained for targeted full-text evaluation, citation chaining, and narrative synthesis

Appendix D

**Table 5:** Descriptive characteristics and performance context of principal human participant studies contributing to the comparative synthesis Across the principal human participant studies contributing to the comparative synthesis, sample sizes ranged from single-participant feasibility studies to studies involving 20 participants, while task sets ranged from three discrete prosthetic hand configurations to 48 hand gestures; some studies instead evaluated continuous movement or control tasks that cannot be expressed as a discrete gesture count. Participant populations, amputation status, sensing configurations, classifiers, and validation procedures also varied substantially. Reported outcomes included within-session cross-validation, cross-trial and cross-day classification, continuous regression, real-time recognition, virtual control, and functional physical prosthesis performance. Measures of dispersion are reported where they were provided by the original publications. Because these outcomes are methodologically and statistically heterogeneous, they were not pooled into an overall accuracy estimate or used to generate a quantitative ranking of the modalities. Companion publications derived from the same or closely related cohorts were considered together where appropriate to avoid treating repeated analyses of the same participants as independent evidence. EMG: electromyography; RPNI: regenerative peripheral nerve interface; FMG: force myography; FSR: force-sensitive resistor; SMG: sonomyography; NIR: near-infrared; OMG: optical myography; LDA: linear discriminant analysis; ANN: artificial neural network; SVM: support vector machine; KNN: K-nearest neighbors; iEMG: integrated electromyography; SD: standard deviation; SEM: standard error of the mean

Modality	Study	Participants	Task/gesture set	Evaluation design	Principal result and reported dispersion
EMG	Waris et al. [13,14]	10 able-bodied+6 individuals with transradial amputation	7 hand motions	Surface, intramuscular, and combined EMG recorded over 7 consecutive days; within-day and between-day evaluation using LDA and multiple classifiers	In the multiday ANN comparison, within-day classification error was 9.12±7.38% for surface EMG, 11.86±7.84% for iEMG, and 6.11±7.46% for combined EMG. Leave-one-day-out between-day error increased to 21.88±4.14%, 29.33±2.58%, and 14.37±3.10%, respectively. The companion analysis similarly demonstrated deterioration in classification performance as the interval between training and testing days increased.
Implanted EMG/RPNI	Vu et al. [46]	2 individuals with transradial amputation	Finger and grasp movements; online prosthetic control and functional multi-grasp task	Longitudinal implanted EMG from regenerative peripheral nerve interfaces and residual innervated muscles	RPNI signal-to-noise ratio remained >15 for up to 276 days in one participant and 1,054 days in the other. One participant maintained >94% real-time prosthetic control accuracy for 604 days without recalibration and completed a multi-grasp coffee-making task with 99% accuracy for 611 days without recalibration.
Optical fiber FMG	Fujiwara and Suzuki [22]	6 healthy participants	9 hand postures	Three optical fiber transducers; ANN; 10-fold cross-validation	Overall sensitivity was 98.4±1.7% and average classification accuracy was 99.7±0.4%.
FSR-FMG	Rehman et al. [23]	6 participants: 3 healthy+3 with transradial amputation	9 upper limb gestures	Seven FSRs; two sensor arrangements; SVM and KNN	For arrangement B, SVM accuracy was 90.0±3.7% and KNN accuracy was 95.4±2.1%. Corresponding arrangement A accuracies were 87.7±3.3% and 91.3±2.3%, respectively.
FSR-FMG	Li et al. [25]	Human participants evaluated across repeated recording sessions	17 finger motions	Thirty-two FSR sensor array; SVM; within-session and intra-wearing cross-session validation	Within-session accuracy exceeded 99%. In intra-wearing cross-session testing, in which the FSR array was not removed or reset, reported accuracies ranged from approximately 79% to 98%. These values should not be interpreted as performance after complete sensor repositioning.
FSR-FMG	Cho et al. [26]	4 male participants with transradial amputation	Up to 11 grips	Residual limb FMG; multiple grip subsets; prosthetic hand feasibility testing	The mean classification accuracy was 41.73±9.63% for all 11 residual limb grips and 62.61±11.54% for the six primary grips. Subdivision according to opposed and non-opposed thumb prosthetic modes improved performance further.
FSR-FMG vs. surface EMG	Jiang et al. [27]	12 able-bodied participants	3 sets totaling 48 hand gestures	Concurrent FMG and surface EMG; cross-validation and cross-trial evaluation	Eight FSRs achieved 91.2% cross-validation and 83.5% cross-trial accuracy compared with 84.6% and 79.1% for surface EMG. Using all 16 FSRs increased FMG performance to 96.7% and 89.4%, respectively.
SMG	Ortenzi et al. [29]	3 intact participants	10 hand postures/grasps and graded grip force levels	B-mode ultrasound; image-derived features; LDA classification	Classification accuracy was approximately 80% for discrete hand postures and grasps but approximately 60% for graded levels of grip force, demonstrating substantial task-dependent performance.
SMG	Lu et al. [31]	10 participants	10 gestures	Wearable A-mode ultrasound; 10-fold offline cross-validation and real-time evaluation	Offline recognition accuracy was 96.92±1.92%. Real-time recognition accuracy was 83.8±6.9%, and action completion rate was 96.0±3.6%.
SMG	Engdahl et al. [54]	8 individuals with upper limb loss: 7 with transradial amputation+1 with congenital limb absence	Participant-specific hand/wrist gesture sets; commonly 5 motions	B-mode SMG; initial exposure, biofeedback/training, and repeated evaluation on different days	Initial cross-validation accuracy was 96.2±5.9% (range: 83.2-100%). In participants evaluated across separate days, the mean accuracy remained stable at 96.9±2.7% in session 1 and 96.7±1.4% in session 2.
SMG	Yin et al. [33]	20 able-bodied participants	6 commonly used gestures	Four-transducer wearable A-mode ultrasound system integrated into a prosthetic hand socket; offline and online testing	The mean online classification accuracy was 91.5±6.4% while wearing the physical prosthesis and 96.5±3.0% without wearing it.
SMG	Yang et al. [34]	4 individuals with transradial amputation	6 finger gestures with simultaneous wrist pronation/supination	Wearable ultrasound array; simultaneous prediction of discrete finger gestures and continuous wrist rotation; comparison with surface EMG	Simultaneous hand gesture and wrist rotation prediction was demonstrated in all four participants. Comparative analyses favored ultrasound over surface EMG under the study conditions, although performance depended on the prediction model and outcome considered.
SMG	Engdahl et al. [35]	1 participant with congenital bilateral limb absence	3 hand configurations: rest, tripod grasp, and index finger point; 3 functional tasks	Real-time SMG control of a physical prosthetic hand across multiple arm positions, 3 hours of use, and simulated donning/doffing	The participant successfully completed all three functional task types and repeated testing without classifier retraining over 3 hours and after simulated donning/doffing. A dynamic classifier trained across four movement patterns achieved offline accuracy of 99.9±0.3% under one socket-loading condition and 100.0±0.0% under the other.
SMG	Yang et al. [55]	10 participants, including 1 participant with limb loss	Simultaneous wrist pronation/supination and hand opening/closing	Self-supervised wearable sonomyography; offline movement estimation and real-time virtual hand control	Offline correlation coefficients for wrist and hand estimates were approximately 0.98 and 0.94 in able-bodied participants and 0.98 and 0.90 in the participant with limb loss. Real-time control of a virtual hand was demonstrated.
SMG	Nazari and Zheng [36]	Offline: 10 healthy participants; real-time: 2 amputees	9 gestures; control of a 6-DOF prosthetic hand	Wireless wearable ultrasound imaging; offline classification followed by real-time physical prosthesis functional testing	All nine gestures were classified with a reported 100% success rate in the offline study. The subsequently developed control system was successfully evaluated in two amputees using a physical 6-DOF prosthetic hand during functional hand tests.
Camera-based OMG	Nissler et al. [37,38]	10 intact participants	4 movement dimensions: thumb rotation, thumb flexion, index finger flexion, and combined flexion of the middle/ring/little fingers	Standard web camera and forearm fiducial markers; regression-based movement estimation using multiple validation procedures	Normalized root mean square error was approximately 0.05-0.22 depending on movement and validation condition. The study reported SD or SEM according to the particular analysis rather than a classification accuracy statistic directly comparable with the other modalities.
NIR-OMG	Miyake et al. [39]	14 healthy participants	5 gripping task types: hand gripper, ball, balloon, palm clenching, and paper crumpling	Fourteen-channel FirstVR NIR wearable sensor; time-series analysis of forearm muscle deformation	The system detected contraction-related muscle deformation patterns and differences in variability and periodicity among the gripping tasks. No prosthetic gesture classification accuracy directly comparable with EMG, FMG, or SMG was reported.
NIR-OMG	Khalikov et al. [41]	8 able-bodied participants+1 participant with limb loss	Gesture-based calibration followed by continuous cursor/target control and gaming tasks	Fifty-channel wearable OMG wristband; neural network-based continuous real-time decoding	All participants successfully used continuous OMG-based control for screen target acquisition and gaming tasks, including the participant with limb loss. Performance was reported using continuous trajectory and task performance measures rather than a conventional discrete gesture classification accuracy.

Appendix E

**Table 6:** Structured methodological appraisal of primary human participant studies contributing to the comparative synthesis Y: Yes; N: No; CT: Can't tell. For quantitative non-randomized studies, the MMAT criteria were as follows: C1, representativeness of participants; C2, appropriateness of outcome and intervention/exposure measurements; C3, completeness of outcome data; C4, accounting for confounding factors; and C5, whether the intervention/exposure occurred as intended. For quantitative descriptive studies, the criteria were as follows: C1, relevance of the sampling strategy; C2, representativeness of the sample; C3, appropriateness of measurements; C4, risk of nonresponse bias; and C5, appropriateness of the statistical analysis. Judgments were based on information reported in the primary publications. Applicability to prosthetic hand users was considered separately because methodological quality within an individual study does not necessarily establish external validity for prosthesis users. No overall numerical quality score was calculated. MMAT: Mixed Methods Appraisal Tool; EMG: electromyography; FMG: force myography; FSR: force-sensitive resistor; SMG: sonomyography; NIR: near-infrared; OMG: optical myography; RPNI: regenerative peripheral nerve interface

Study	Modality/study context	MMAT category	C1	C2	C3	C4	C5	Main limitation relevant to this review
Waris et al. [13,14]	Surface/intramuscular EMG; longitudinal seven-day cohort	Quantitative non-randomized	N	Y	N	Y	Y	Small, predominantly male cohort; two recruited amputees withdrew; laboratory classification rather than unrestricted everyday prosthesis use
Tobias et al. [18]	Commercial myoelectric prosthesis; functional training	Quantitative non-randomized	N	Y	Y	N	Y	Single participant with congenital transradial limb difference; uncontrolled pre/post feasibility design
Fujiwara and Suzuki [22]	Optical fiber FMG; nine-posture classification	Quantitative descriptive	CT	N	Y	CT	Y	Six healthy participants; controlled laboratory postures; no direct prosthesis user validation
Rehman et al. [23]	FSR-FMG; comparison of two armband arrangements	Quantitative non-randomized	N	Y	Y	CT	Y	Six participants, including only three individuals with transradial amputation; controlled laboratory gesture protocol
Li et al. [25]	FSR-FMG; 17 finger motions and repeated-session evaluation	Quantitative descriptive	CT	CT	Y	CT	Y	Cross-session results were obtained without complete removal and repositioning of the FSR array, limiting inference regarding repeated donning
Cho et al. [26]	FSR-FMG; residual limb grip recognition and prosthetic hand feasibility	Quantitative descriptive	Y	N	Y	Y	Y	Four male participants with transradial amputation; small and demographically limited sample
Jiang et al. [27]	FMG versus surface EMG; 48 hand gestures	Quantitative non-randomized	N	Y	Y	Y	Y	Twelve able-bodied participants; laboratory comparison without validation in prosthesis users
Sikdar et al. [28]	Wearable ultrasound/SMG; individual finger movements	Quantitative descriptive	CT	N	Y	CT	Y	Ten healthy volunteers; potential prosthetic control applicability rather than direct amputee prosthesis testing
Ortenzi et al. [29]	B-mode SMG; posture/grasp and grip force classification	Quantitative descriptive	CT	N	Y	Y	Y	Only three intact participants; controlled probe positioning and laboratory tasks
Castellini et al. [30]	B-mode ultrasound; finger position estimation	Quantitative descriptive	CT	N	Y	CT	Y	Six able-bodied participants; prosthetic application proposed rather than directly evaluated
Lu et al. [31]	Wearable A-mode SMG; offline and real-time gesture recognition	Quantitative descriptive	CT	CT	Y	CT	Y	Ten-participant controlled gesture protocol; real-time recognition demonstrated without direct physical hand prosthesis testing
Engdahl et al. [54]	SMG classification in individuals with upper limb loss; initial exposure, training, and repeated-day testing	Quantitative descriptive	Y	N	Y	CT	Y	Eight participants with upper limb loss; not all participants completed every experimental phase; laboratory classification rather than direct physical prosthesis use
Yin et al. [33]	Wearable A-mode SMG integrated into a prosthetic hand socket	Quantitative descriptive	CT	N	Y	CT	Y	Twenty able-bodied participants; physical prosthesis configuration was evaluated, but no individuals with limb loss were included
Yang et al. [34]	Wearable ultrasound versus surface EMG in individuals with transradial amputation	Quantitative non-randomized	N	Y	Y	CT	Y	Only four participants with transradial amputation; controlled experimental task and limited sample size
Engdahl et al. [35]	Real-time SMG control of a physical prosthetic hand during functional tasks	Quantitative descriptive	Y	N	Y	Y	CT	Single-participant case study; strong functional feasibility evidence but very limited generalizability
Yang et al. [55]	Self-supervised SMG; simultaneous wrist/hand estimation and virtual hand control	Quantitative non-randomized	N	Y	Y	CT	Y	Ten participants but only one participant with limb loss; online evaluation used a virtual rather than physical prosthetic hand
Nazari and Zheng [36]	Wireless wearable SMG; offline classification and real-time 6-DOF prosthetic hand control	Quantitative descriptive	CT	N	Y	CT	Y	Offline performance evaluated in 10 healthy participants, whereas real-time physical prosthesis evaluation included only two amputees
Nissler et al. [37,38]	Camera-based OMG; finger motion estimation	Quantitative descriptive	CT	N	Y	CT	Y	Ten intact participants; fixed camera, stabilized forearm, and fiducial markers; no validation in individuals with amputation
Miyake et al. [39]	NIR-OMG; wearable muscle deformation monitoring during gripping tasks	Quantitative descriptive	CT	N	Y	CT	Y	Fourteen healthy participants; study evaluated muscle deformation monitoring rather than direct prosthetic hand control
Khalikov et al. [41]	Wearable NIR-OMG; continuous real-time human-machine control	Quantitative descriptive	CT	N	Y	CT	Y	Eight able-bodied participants and only one participant with limb loss; continuous cursor/game control rather than long-term control of a physical prosthetic hand
Vu et al. [46]	Implanted EMG/RPNI; longitudinal prosthetic control	Quantitative non-randomized	N	Y	N	CT	Y	Only two individuals with transradial amputation; detailed sustained online decoder and functional evaluation was predominantly demonstrated in one participant

## References

[1] Yuan B, Hu D, Gu S, Xiao S, Song F (2023). The global burden of traumatic amputation in 204 countries and territories. Front Public Health.

[2] Biddiss EA, Chau TT (2007). Upper limb prosthesis use and abandonment: a survey of the last 25 years. Prosthet Orthot Int.

[3] Farina D, Aszmann O (2014). Bionic limbs: clinical reality and academic promises. Sci Transl Med.

[4] Lucaccini LF, Kaiser PK, Lyman J (1966). The French electric hand: some observations and conclusions. Bull Prosthet Res.

[5] Wang Y, Tian Y, She H, Jiang Y, Yokoi H, Liu Y (2022). Design of an effective prosthetic hand system for adaptive grasping with the control of myoelectric pattern recognition approach. Micromachines (Basel).

[6] Hong QN, Fàbregues S, Bartlett G (2018). The Mixed Methods Appraisal Tool (MMAT) version 2018 for information professionals and researchers. Educ Inf.

[7] Ziegler-Graham K, MacKenzie EJ, Ephraim PL, Travison TG, Brookmeyer R (2008). Estimating the prevalence of limb loss in the United States: 2005 to 2050. Arch Phys Med Rehabil.

[8] Cordella F, Ciancio AL, Sacchetti R, Davalli A, Cutti AG, Guglielmelli E, Zollo L (2016). Literature review on needs of upper limb prosthesis users. Front Neurosci.

[9] Kuiken TA, Li G, Lock BA, Lipschutz RD, Miller LA, Stubblefield KA, Englehart KB (2009). Targeted muscle reinnervation for real-time myoelectric control of multifunction artificial arms. JAMA.

[10] Farina D, Jiang N, Rehbaum H, Holobar A, Graimann B, Dietl H, Aszmann OC (2014). The extraction of neural information from the surface EMG for the control of upper-limb prostheses: emerging avenues and challenges. IEEE Trans Neural Syst Rehabil Eng.

[11] Disselhorst-Klug C, Williams S (2020). Surface electromyography meets biomechanics: correct interpretation of sEMG-signals in neuro-rehabilitation needs biomechanical input. Front Neurol.

[12] Antfolk C, D'Alonzo M, Rosén B, Lundborg G, Sebelius F, Cipriani C (2013). Sensory feedback in upper limb prosthetics. Expert Rev Med Devices.

[13] Waris A, Niazi IK, Jamil M, Englehart K, Jensen W, Kamavuako EN (2019). Multiday evaluation of techniques for EMG-based classification of hand motions. IEEE J Biomed Health Inform.

[14] Waris A, Niazi IK, Jamil M (2018). The effect of time on EMG classification of hand motions in able-bodied and transradial amputees. J Electromyogr Kinesiol.

[15] De Luca CJ (1997). The use of surface electromyography in biomechanics. J Appl Biomech.

[16] Clancy EA, Morin EL, Merletti R (2002). Sampling, noise-reduction and amplitude estimation issues in surface electromyography. J Electromyogr Kinesiol.

[17] Jiang N, Dosen S, Müller KR, Farina D (2012). Myoelectric control of artificial limbs-is there a need to change focus?. IEEE Signal Process Mag.

[18] Tobias M, Okhuoya O, Ancel J, Intintoli M, Thompson LA, Chen J (2025). Evaluation of a myoelectrical arm for transradial amputation in functional activities. Appl Sci.

[19] Xiao ZG, Menon C (2019). A review of force myography research and development. Sensors (Basel).

[20] Abboudi RL, Glass CA, Newby NA, Flint JA, Craelius W (1999). A biomimetic controller for a multifinger prosthesis. IEEE Trans Rehabil Eng.

[21] Sherif O, Bassuoni MM, Mehrez O (2024). A survey on the state of the art of force myography technique (FMG): analysis and assessment. Med Biol Eng Comput.

[22] Fujiwara E, Suzuki CK (2018). Optical fiber force myography sensor for identification of hand postures. J Sens.

[23] Rehman MU, Shah K, Haq IU, Iqbal S, Ismail MA (2023). A wearable force myography-based armband for recognition of upper limb gestures. Sensors (Basel).

[24] Xiao ZG, Menon C (2019). An investigation on the sampling frequency of the upper-limb force myographic signals. Sensors (Basel).

[25] Li N, Yang D, Jiang L, Liu H, Cai H (2012). Combined use of FSR sensor array and SVM classifier for finger motion recognition based on pressure distribution map. J Bionic Eng.

[26] Cho E, Chen R, Merhi LK, Xiao Z, Pousett B, Menon C (2016). Force myography to control robotic upper extremity prostheses: a feasibility study. Front Bioeng Biotechnol.

[27] Jiang X, Merhi LK, Xiao ZG, Menon C (2017). Exploration of force myography and surface electromyography in hand gesture classification. Med Eng Phys.

[28] Sikdar S, Rangwala H, Eastlake EB (2014). Novel method for predicting dexterous individual finger movements by imaging muscle activity using a wearable ultrasonic system. IEEE Trans Neural Syst Rehabil Eng.

[29] Ortenzi V, Tarantino S, Castellini C, Cipriani C (2015). Ultrasound imaging for hand prosthesis control: a comparative study of features and classification methods. 2015 IEEE International Conference on Rehabilitation Robotics (ICORR).

[30] Castellini C, Passig G, Zarka E (2012). Using ultrasound images of the forearm to predict finger positions. IEEE Trans Neural Syst Rehabil Eng.

[31] Lu Z, Cai S, Chen B, Liu Z, Guo L, Yao L (2022). Wearable real-time gesture recognition scheme based on A-mode ultrasound. IEEE Trans Neural Syst Rehabil Eng.

[32] Engdahl SM, Acuña SA, Kaliki RR, Sikdar S (2024). Sonomyography for control of upper-limb prostheses: current state and future directions. J Prosthet Orthot.

[33] Yin Z, Chen H, Yang X, Liu Y, Zhang N, Meng J, Liu H (2022). A wearable ultrasound interface for prosthetic hand control. IEEE J Biomed Health Inform.

[34] Yang X, Liu Y, Yin Z, Wang P, Deng P, Zhao Z, Liu H (2022). Simultaneous prediction of wrist and hand motions via wearable ultrasound sensing for natural control of hand prostheses. IEEE Trans Neural Syst Rehabil Eng.

[35] Engdahl SM, Acuña SA, King EL, Bashatah A, Sikdar S (2022). First demonstration of functional task performance using a sonomyographic prosthesis: a case study. Front Bioeng Biotechnol.

[36] Nazari V, Zheng YP (2025). A highly efficient HMI algorithm for controlling a multi-degree-of-freedom prosthetic hand using sonomyography. Sensors (Basel).

[37] Nissler C, Mouriki N, Castellini C, Belagiannis V, Navab N (2015). OMG: introducing optical myography as a new human machine interface for hand amputees. 2015 IEEE International Conference on Rehabilitation Robotics (ICORR).

[38] Nissler C, Mouriki N, Castellini C (2016). Optical myography: detecting finger movements by looking at the forearm. Front Neurorobot.

[39] Miyake T, Minakuchi T, Sato S, Okubo C, Yanagihara D, Tamaki E (2024). Optical myography-based sensing methodology of application of random loads to muscles during hand-gripping training. Sensors (Basel).

[40] Zhuwawu SS, Owen M, Bifet A, Dwivedi A (2026). Muscle myography for human-machine interfaces: a review of sensing modalities and control interfaces. Front Bioeng Biotechnol.

[41] Khalikov R, Soghoyan G, Sintsov M, Lebedev M (2026). Wearable optomyography enables continuous neuroprosthetic control. Sci Rep.

[42] Phinyomark A, Phukpattaranont P, Limsakul C (2012). Feature reduction and selection for EMG signal classification. Expert Syst Appl.

[43] Englehart K, Hudgins B (2003). A robust, real-time control scheme for multifunction myoelectric control. IEEE Trans Biomed Eng.

[44] Atzori M, Cognolato M, Müller H (2016). Deep learning with convolutional neural networks applied to electromyography data: a resource for the classification of movements for prosthetic hands. Front Neurorobot.

[45] He J, Zhang D, Jiang N, Sheng X, Farina D, Zhu X (2015). User adaptation in long-term, open-loop myoelectric training: implications for EMG pattern recognition in prosthesis control. J Neural Eng.

[46] Vu PP, Vaskov AK, Lee C (2023). Long-term upper-extremity prosthetic control using regenerative peripheral nerve interfaces and implanted EMG electrodes. J Neural Eng.

[47] Ribas Neto A, Fajardo J, da Silva WH (2021). Design of tendon-actuated robotic glove integrated with optical fiber force myography sensor. Automation.

[48] Navarro X, Krueger TB, Lago N, Micera S, Stieglitz T, Dario P (2005). A critical review of interfaces with the peripheral nervous system for the control of neuroprostheses and hybrid bionic systems. J Peripher Nerv Syst.

[49] Chopra S, Emran TB (2024). Advances in AI-based prosthetics development: editorial. Int J Surg.

[50] Kim DH, Lu N, Ma R (2011). Epidermal electronics. Science.

[51] Shimizu Y, Mori T, Yoshikawa K (2024). Developing a novel prosthetic hand with wireless wearable sensor technology based on user perspectives: a pilot study. Sensors (Basel).

[52] Tarantino S, Clemente F, Barone D, Controzzi M, Cipriani C (2017). The myokinetic control interface: tracking implanted magnets as a means for prosthetic control. Sci Rep.

[53] Moradi A, Rafiei H, Daliri M, Akbarzadeh-T MR, Akbarzadeh A, Naddaf-Sh AM, Naddaf-Sh S (2022). Clinical implementation of a bionic hand controlled with kineticomyographic signals. Sci Rep.

[54] Engdahl S, Dhawan A, Bashatah A (2022). Classification performance and feature space characteristics in individuals with upper limb loss using sonomyography. IEEE J Transl Eng Health Med.

[55] Yang X, Yin Z, Sheng Y, Farina D, Liu H (2025). Self-supervised learning for intuitive control of prosthetic hand movements via sonomyography. IEEE Trans Cybern.

